# Linear and Nonlinear Optical Properties of All-*cis* and All-*trans* Poly(*p*-phenylenevinylene)

**DOI:** 10.1021/acs.jpcc.3c07082

**Published:** 2024-02-02

**Authors:** Haraprasad Mandal, Olusayo J. Ogunyemi, Jake L. Nicholson, Meghan E. Orr, Remy F. Lalisse, Ángel Rentería-Gómez, Achyut R. Gogoi, Osvaldo Gutierrez, Quentin Michaudel, Theodore Goodson

**Affiliations:** † Department of Chemistry, University of Michigan, Ann Arbor, Michigan 48109, United States; ‡ Department of Macromolecular Science & Engineering, University of Michigan, Ann Arbor, Michigan 48109, United States; § Department of Chemistry, Texas A&M University, College Station, Texas 77843, United States; ∥ Department of Materials Science and Engineering, Texas A&M University, College Station, Texas 77843, United States

## Abstract

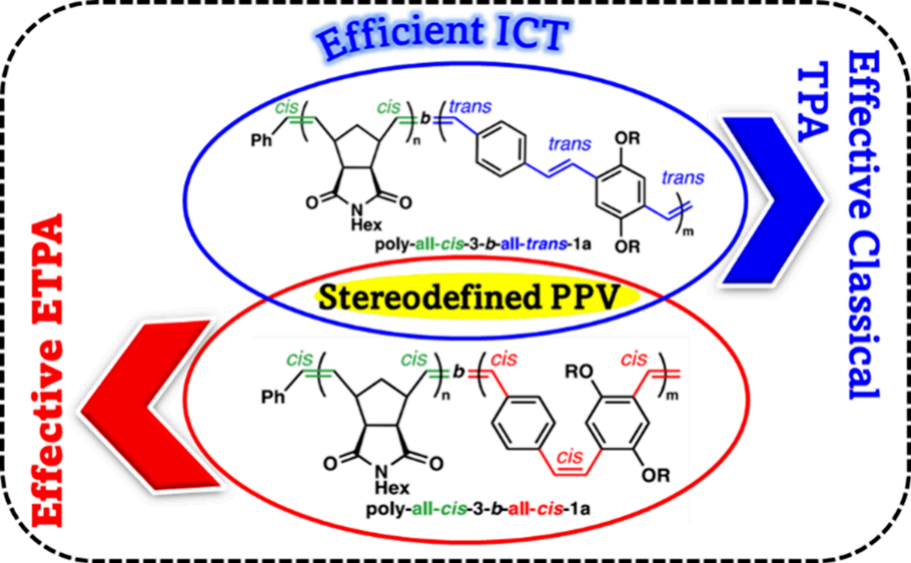

Poly(*p*-phenylenevinylene) (PPV) is a staple of the family of conjugated polymers with desirable optoelectronic properties for applications including light-emitting diodes (LEDs) and photovoltaic devices. Although the significant impact of olefin geometry on the steady-state optical properties of PPVs has been extensively studied, PPVs with precise stereochemistry have yet to be investigated using nonlinear optical spectroscopy for quantum sensing, as well as light harvesting for biological applications. Herein, we report our investigation of the influence of olefin stereochemistry on both linear and nonlinear optical properties through the synthesis of all-*cis* and all-*trans* PPV copolymers. We performed two-photon absorption (TPA) using a classical and entangled light source and compared both classical TPA and entangled two-photon absorption (ETPA) cross sections of these stereodefined PPVs. Whereas the TPA cross section of the all-*trans* PPV was expectedly higher than that of all-*cis* PPV, presumably because of the larger transition dipole moment, the opposite trend was measured via ETPA, with the all-*cis* PPV exhibiting the highest ETPA cross section. DFT calculations suggest that this difference might stem from the interaction of entangled photons with lower-lying electronic states in the all-*cis* PPV variant. Additionally, we explored the photoinduced processes for both *cis* and *trans* PPVs through time-resolved fluorescence upconversion and femtosecond transient absorption techniques. This study revealed that the sensitivity of PPVs in two-photon absorption varies with classical versus quantum light and can be modulated through the control of the geometry of the repeating alkenes, which is a key stepping stone toward their use in quantum sensing, bioimaging, and the design of polymer-based light-harvesting systems.

## Introduction

Over the years, there has been increased interest in utilizing conjugated polymers with unique electroluminescence properties for applications in organic light-emitting diodes (OLEDs) in display technologies, as organic semiconductors in photovoltaic cells for energy conversion and in energy storage devices, and as solid-state laser materials.^1,2^ Among these, the properties of poly(*p*-phenylenevinylene) (PPV) and its derivatives have been extensively studied, which have paved the way for their use in optical devices and bulk-heterojunction solar cells.^3−8^ PPVs consist of alternating *p-*phenylene and vinylene subunits with higher stability compared to polyacetylene and more flexibility compared to polyphenylene.^2,4^ The parent unsubstituted PPV has a band gap of 2.5 eV with a broad spectrum in the visible region, both features that can be modulated through structural derivatization, which is crucial for implementation into photoluminescence devices.^9^ The structure and conformation of conjugated polymers are known to determine the fluorescence lifetime, photoluminescence efficiency, as well as electron and charge mobilities in resulting devices. For example, the alkenes in the PPV backbone can exhibit either *cis* or *trans* configurations, which dictates the overall chain conformation. A high *trans* content typically results in rodlike structures that favor π–π stacking and therefore aggregation, whereas a high *cis* content leads to more coiled and twisted structures with reduced conjugation length.^10−12^ Both π–π stacking and conjugation length drastically affect the charge transport mobility of the polymer, along with the conformation of its backbone. It has been observed that polymers with linear backbones have higher charge transfer properties than polymers with flexible or wavelike backbones.^13,14^ The modularity of synthetic methods^1,2^ to modify the PPV scaffold via copolymerization or post polymerization functionalization has permitted the synthesis of numerous derivatives with improved conjugation length, solubility, and packing along the polymer backbone through the addition of nonconjugated carbon bridges or conjugated π-linkers. Efficient packing leads to improved optical, charge transport, and sensing properties.^15−19^ In addition to being pursued for applications discussed above (OLEDs, photovoltaics, etc.), PPVs also exhibit biocompatibility, low toxicity, and high fluorescence quantum yields, making them promising fluorophores for bioimaging applications.^20^ For example, in a recent study by Junkers and co-workers, amphiphilic PPV copolymers were synthesized to form fluorescent micelles for drug delivery that were imaged using classical light and laser scanning confocal microscopy.^21^

**Figure 1:**
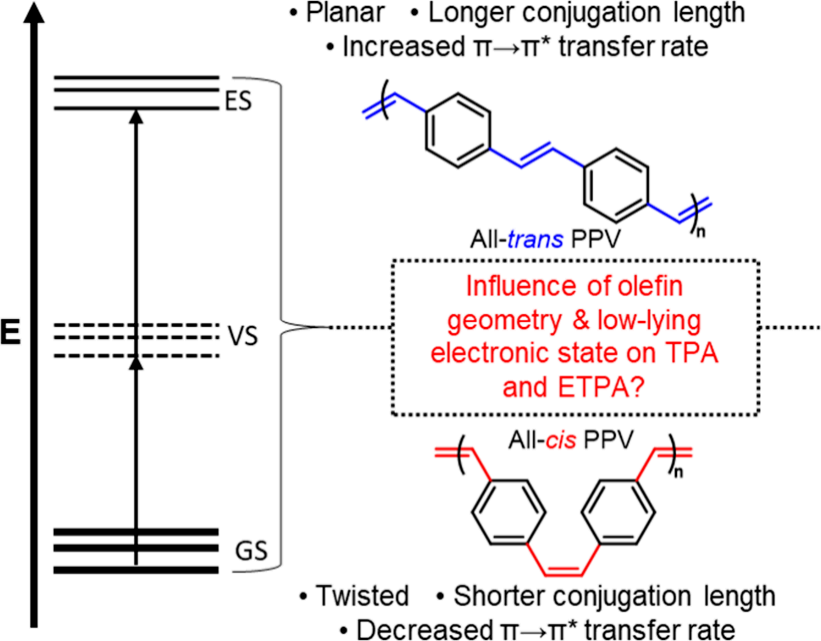
Schematic energy diagram of a two-photon absorption process happening from the ground state (GS) through the virtual state (VS) to the two-photon excited state (ES). A summary of property differences between all-*trans* PPV and all-*cis* PPV is also shown.

The use of PPV materials in nonlinear optical (NLO) applications is especially intriguing because NLO effects can be exploited for optical communication, frequency doubling and tripling, and photorefractive effect.^22,23^ Although several studies have allowed the identification of the structural parameters of organic materials responsible for NLO,^24−26^ it remains crucial to design new organic chromophores to further understand and optimize NLO phenomena prior to their technological implementation. Of particular interest, some organic materials have been shown recently to exhibit NLO phenomena sensitive to the use of entangled photons.^27−30^ The two entangled photons generated by the process of spontaneous parametric downconversion (SPDC) share a coupled eigenstate where the properties of each of the photons cannot be isolated separately.^31,32^ Unlike classical two-photon absorption (TPA), strong temporal and spatial correlations of the entangled photon pair lead to the simultaneous absorption of the two photons (Figure 1) at the condition that the absorption occurs within a time window known as the entanglement time, *T*_e_.^29,33^ This phenomenon is known as entangled two-photon absorption (ETPA). Unlike classical TPA where the TPA has a quadratic dependence on input photon flux, studies have shown that the ETPA rate exhibits a linear dependence on the excitation flux.^28,30^ This linear dependency is dominant only when the flux intensities are sufficiently low.^34,35^ Entangled two-photon absorption therefore allows for improved resolution and sensitivity at lower excitation flux compared to classical TPA, which is promising for applications in quantum sensing, optical communication, and bioimaging.^32^ Because of their synthetic versatility, the PPV family offers many opportunities to investigate the relationships between the precise molecular structure of organic materials and NLO behaviors with both classical and entangled light.

Accessing PPVs with >99% *cis* alkenes has been a longstanding synthetic challenge due to the thermodynamic bias for the *trans* vinylene phenylene motif,^1,2^ which has limited nonlinear spectroscopy studies to PPVs with mostly *trans* alkenes or *cis*/*trans* mixtures. For example, Lavrentiev et al. theoretically investigated the low-lying electronic structure of PPV,^36^ whereas Guo and Shih extensively studied the low-lying two-photon excited states in TPA spectroscopy with substituted and unsubstituted PPVs.^37^ Ghosh et al. explored the exciton dynamics and formation mechanism of polymeric nanostructures based on poly(2-methoxy-5-(2′-ethylhexyloxy)-*p*-phenylenevinylene) (MEH-PPV) by using a femtosecond Ti:sapphire pulse laser as the light source.^38^ In addition, De Boni et al. reported the degenerate nonlinear absorption spectrum of MEH-PPV in chloroform using a femtosecond *Z*-scan technique.^39^ The TPA cross-section spectrum of MEH-PPV was also determined by Oliveira et al.^40^ using a white-light continuum (WLC) *Z*-scan technique.

Herein, we discuss our investigation of the interaction of two diblock copolymers containing either an all-*cis* or an all-*trans* PPV segment (Figure 1) with both classical and entangled light in two-photon absorption. The diblock copolymers were synthesized via a stereoselective ring-opening metathesis polymerization (ROMP) of a paracyclophane-1,9-diene monomer used in combination with a norbornene imide derivative. We then measured the TPA and ETPA of both diblock copolymers and explored their photoinduced relaxation processes by time-resolved fluorescence upconversion and femtosecond transient absorption techniques to investigate their intrachain charge transfer nature.

## Experimental Section

### Synthesis of Monomers and Polymers

Monomers **1** and **2** were synthesized according to literature procedures^41,42^ (see the Supporting Information for more details). To synthesize all-*cis* block copolymer poly-*cis*-**1**-*b*-*cis*-**2**, monomer **1** (73.3 mg, 296.4 μmol, 30 equiv) was first measured into a reaction vial charged with a stir bar inside a nitrogen-filled glovebox. The ruthenium catalyst **Ru-St** (8.6 mg, 9.9 μmol, 1 equiv) dissolved in deoxygenated THF (0.10 mL) was then added to the vial, and the mixture was stirred for 1 h at room temperature. Into a second reaction vial containing monomer **2** (52.2 mg, 113.3 μmol, 15 equiv) and a stir bar, 0.08 mL of the mixture was then transferred. The reaction was run in the dark at 40 °C for 2 h before cooling to room temperature and quenching with excess ethyl vinyl ether (0.1 mL). After leaving for an additional 30 min at room temperature, two cycles of precipitation with addition of methanol, centrifugation, and decantation afforded the isolated polymer. After being wrapped in aluminum foil and concentrated under reduced pressure, 23 mg of poly-*cis*-**1**-*b*-*cis*-**2** was then dissolved in 23 mL of DCM and irradiated for 1 h at room temperature using two 350 nm UV lamps placed 1 cm away from the sample to afford poly-*cis*-**1**-*b*-*trans*-**2**. The sample was wrapped in aluminum foil and concentrated under reduced pressure. All polymer samples were fully dried, kept wrapped in aluminum foil, and stored under an inert atmosphere at −20 °C.

### Entangled Two-Photon Absorption

The entangled two-photon absorption experimental setup that had been previously described was used for the purpose of this study.^43^ Type I noncollinear spontaneous parametric downconversion (SPDC) was generated by using a type I BBO crystal with a portable CW laser. The 405 nm light generated from the CW laser goes through a variable neutral density filter, which is used to adjust the pump power into the BBO crystal, resulting in SPDC and entangled photon production. After passing through the sample in the cuvette, the entangled photons are refocused via a band-pass filter with a planoconvex lens before entering the avalanche photodiode (APD). A final band-pass filter was introduced to remove any extra wavelength of light other than the entangled light that may have entered the system because of entangled photon (EP) interactions with the material that resulted in fluorescence or emission.

To measure the EP transmission rate using an APD, the ETPA experiment started by introducing pure chloroform into the cuvette. The variable neutral density filter (NDF) was used to optimize the power of the laser going into the BBO crystal, and as a result, the intensity of the entangled light that interacts with the sample could be controlled. To calibrate the stage, a maximum of 5.5 × 10^6^ uncorrected photon counts per second (cps) was targeted. Once this maximum input photon rate was set, the ETPA scan was taken for a total of 10 input photon rate values. After a baseline absorbance for the pure solvent was found, the solvent was replaced with a diluted PPV sample dissolved in chloroform so that EP transmission could be measured again. The loss in EP transmission seen between the sample solution and the pure solvent is due to ETPA absorption. At least three measurements were taken of each PPV sample to ensure the best signal-to-noise ratio. Because ETPA was performed at such a low photon input intensity, it usually took more than one measurement to get a stable set of readings.

## Result and Discussion

### Design and Synthesis of PPVs for This Study

**Figure 2:**
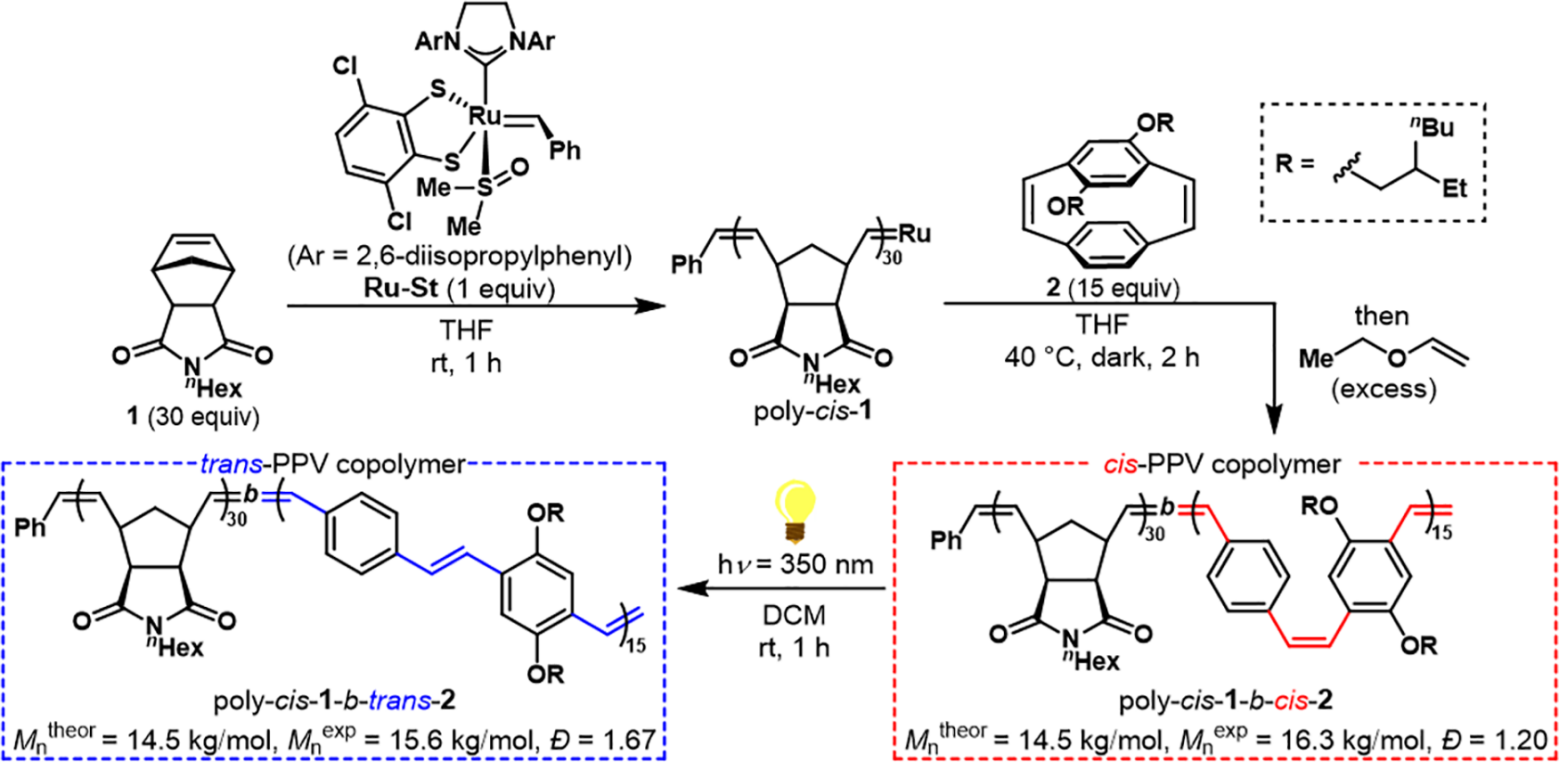
Synthesis of diblock copolymers containing either an all-*cis* or an all-*trans* PPV segment: sequential stereoretentive ROMP of monomers **1** and **2** using catalyst **Ru-St** delivered poly*-cis*-**1***-b*-*cis*-**2**; subsequent PPV-selective photoisomerization afforded poly*-cis*-**1***-b*-*trans*-**2**.

To access stereodefined PPVs with either >99% *cis* or >99% *trans* alkenes, we relied on a two-step approach: First, stereoretentive ring-opening metathesis polymerization (ROMP)^44−46^ of a paracyclophane-1,9-diene monomer^47−51^ would deliver an all-*cis* PPV segment, and then selective photoisomerization of the PPV block would provide the all-*trans* congener.^11^ To optimize the solubility in organic solvents of both *cis* and *trans* PPVs, we designed block copolymers containing a solubilizing poly(norbornene imide) segment stitched to the stereodefined PPV. Additionally, 2-ethylhexyloxy side chains that are known to impart high solubility to conjugated polymers were selected as substituents of the PPV.^52^ Adapting a protocol recently developed by Michaudel and co-workers,^10,53^ two stereodefined copolymers were synthesized and isolated for two-photon absorption spectroscopy (Figure 2). The all-*cis* poly(norbornene imide) segment was first synthesized using a 30:1 ratio of monomer **1** to the **Ru-St** initiator. Chain extension using 15 equiv of monomer **2** followed by termination with excess ethyl vinyl ether afforded the all-*cis* block copolymer poly-*cis*-**1**-*b*-*cis*-**2**. The *cis*-selectivity of the polymerization (>99%) was assessed using NMR spectroscopy (Figures S1 and S2). Size exclusion chromatography (SEC) analysis revealed a narrow dispersity (*Đ* = 1.20) for poly-*cis*-**1**-*b*-*cis*-**2** and good agreement between the theoretical and experimental number-average molecular weight (Figure S5 and Table S1). Photoisomerization of the PPV block under UV light (350 nm) delivered the *trans* PPV variant poly-*cis*-**1**-*b*-*trans*-**2** with exquisite selectivity (>99% *trans* PPV) and no isomerization of the poly(norbornene imide) block as shown by NMR spectroscopy (Figures S3 and S4).

### Steady-State Absorption and Emission

**Figure 3:**
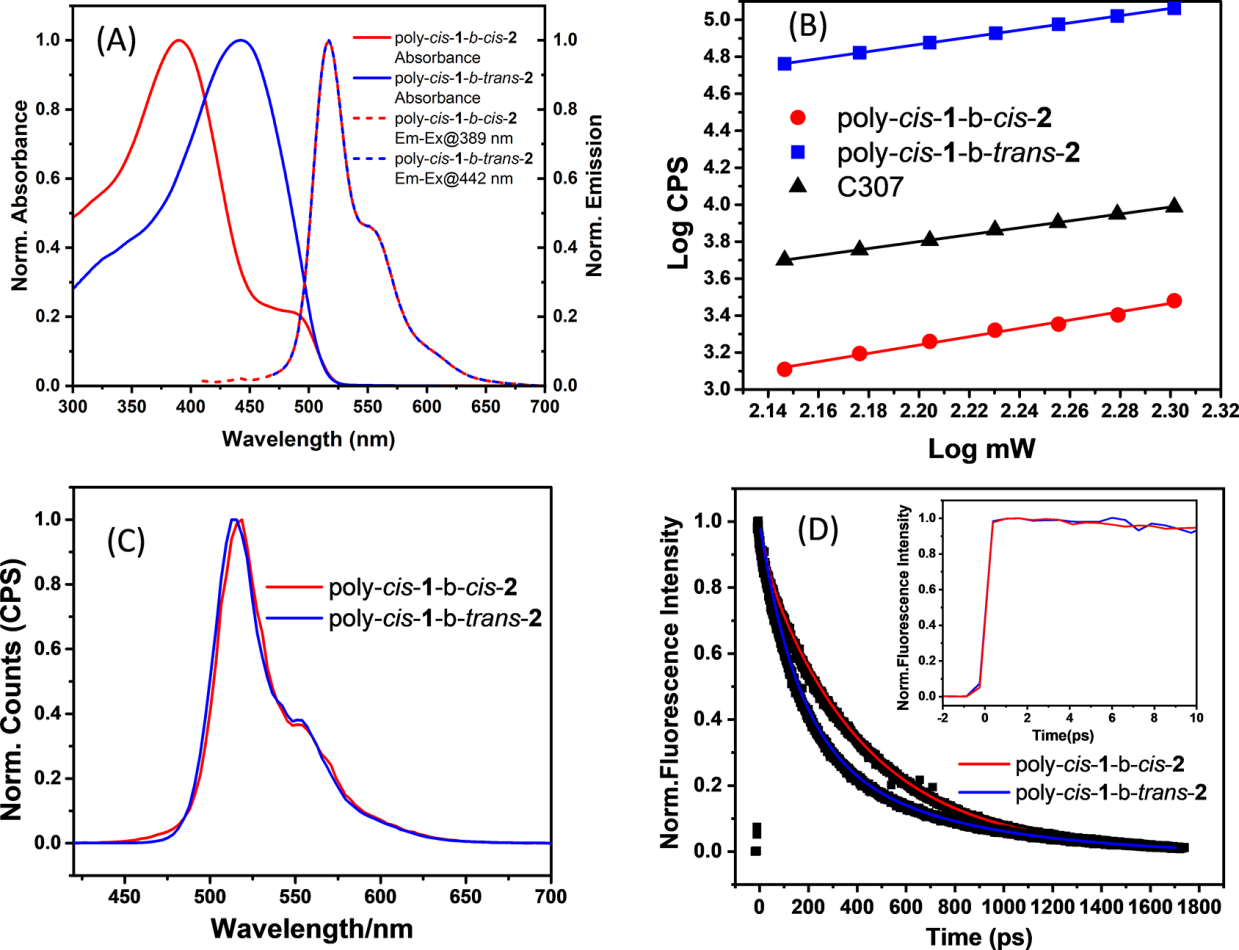
Normalized steady-state absorption (*C* = 25.0 and 12.5 μg/mL, respectively) and emission (*C* = 4.2 and 1.6 ng/mL, respectively) spectra of poly-*cis*-**1**-*b-cis*-**2** and poly-*cis*-**1**-*b*-*trans*-**2** in chloroform (A). Excitation power dependent classical TPA fluorescence (at 520 nm) (B) and two-photon excited fluorescence spectra (C) of the investigated compounds by excitation at 800 nm. Fluorescence decay traces of poly-*cis*-**1**-*b*-*cis*-**2** and poly-*cis*-**1**-*b*-*trans*-**2** at the emissive wavelength of 517 nm after excitation at 400 nm (D). The inset of the panel D shows the emissive decay traces in the 10 ps time window.

UV–vis absorption measurements for poly-*cis*-**1**-*b*-*cis*-**2** and poly-*cis*-**1**-*b*-*trans*-**2** were carried out in chloroform at room temperature with maximum π–π* absorptions of 389 and 442 nm, respectively, indicating a red-shift after photoisomerization consistent with previous studies^10,53−55^ (Figure 3A). Interestingly, emission spectra for poly-*cis*-**1**-*b*-*cis*-**2** and poly-*cis*-**1**-*b*-*trans*-**2** collected following excitation at 389 and 442 nm, respectively, were almost identical. An emission maximum was observed at 517 nm in both spectra. In addition, another weak band at the region of 555 nm for both PPV samples was observed and is consistent with a previous report.^10^ Relative quantum yields were determined, and poly-*cis*-**1**-*b*-*trans*-**2** was found to have a quantum yield 20% higher than poly-*cis*-**1**-*b*-*cis*-**2**. All steady-state results are summarized in Table 1. The narrower optical band gap and higher quantum yield observed for poly-*cis*-**1**-*b*-*trans*-**2** suggest a more efficient intramolecular charge transfer (ICT)^56−59^ compared to poly-*cis*-**1**-*b*-*cis*-**2**.

**Table 1:** Steady-State, Classical, and Entangled Two-Photon Absorption Results of Poly-*cis*-**1***-b-cis*-**2** and Poly-*cis*-**1-***b*-*trans*-**2** in Chloroform

samples	absorption (nm)	emission (nm)	Φ_fl_ (%)	TPA cross section (±0.3) (GM)	ETPA cross section (±0.05) (cm^2^/molecule)
poly-*cis*-1-*b*-*cis*-2	389	517, 555	36.3	11.4	5.0 × 10^–18^
poly-*cis*-1-*b*-*trans*-2	442	517, 555	56.5	1490	2.2 × 10^–18^

### Classical Two-Photon Absorption

The nonlinear optical properties of poly-*cis*-**1**-*b*-*cis*-**2** and poly-*cis*-**1**-*b*-*trans*-**2** were then analyzed using classical TPA (λ_exc_ = 800 nm) with induced fluorescence detection to gain insight into the charge transfer character. It has been observed that the transition probability of TPA increases with increasing ICT character.^60^ The logarithmic plot of the intensity (counts per second) versus the laser power (mW) shows a linear fit with a slope value of about 2 for both PPV samples (Figure 3B). This indicates that a two-photon excitation process is allowed in both poly-*cis*-**1**-*b*-*cis*-**2** and poly-*cis*-**1**-*b*-*trans*-**2**. Figure 3B shows the power dependent two-photon excited fluorescence spectra, whereas Figure 3C depicts the two-photon emission, which correlates well with the steady-state emission spectra. The slope values obtained from the power-dependence logarithm plot were used to calculate the two-photon absorption cross section.^61^ The TPA cross sections were calculated using the following equation:
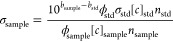
1where ϕ is the emission quantum yield, *n* is the solvent refractive index, *b* is the intercept in the linear fit of the quadratic power dependence, and [*c*] is the concentration. The two-photon photoluminescence spectra were measured by exciting the sample at 800 nm and collecting the photoluminescence at 520 nm, the two-photon excited fluorescence maxima (Figure 3C). In all cases, the two-photon excited emission spectra are very similar to the one-photon steady-state emission spectra (Figure 3A). The TPA cross-section values for both poly-*cis*-**1**-*b*-*cis*-**2** and poly-*cis*-**1**-*b*-*trans*-**2** at the same concentration (3.97 × 10^–6^ M) were found to be 11.4 and 1490 GM (1 Göppert-Mayer = 10^–50^ cm^4^ s photon^–1^), respectively, as listed in Table 1. The TPA cross section for poly-*cis*-**1**-*b*-*trans*-**2** was found to be much higher than that for poly-*cis*-**1**-*b*-*cis*-**2**, indicating the more efficient ICT nature of poly-*cis*-**1**-*b*-*trans*-**2** that is useful for OLEDs, two-photon imaging, and photovoltaic applications. This higher TPA cross section was ascribed to the increased conjugation length and higher π–π interaction present in poly-*cis*-**1**-*b*-*trans*-**2** as a result of the more rodlike structure of the *trans* PPV segment, which allows for more effective charge mobility along the polymer backbone. To better understand the TPA cross section in terms of dipole moments and the different TPA mechanisms, the random (classical) TPA expression can also be written as^60^
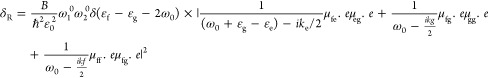
2

The first term in eq 2 describes the TPA through an intermediate level. Alternatively, the second and third terms in eq 2 describe TPA through the transition dipole pathway, where the ground and final states are strongly coupled to each other. Therefore, in the transition dipole pathway, intermediate states are not involved. Chouk et al. described that the transition dipole-moment of *trans*-PPV is three times greater due to the higher electron mobility created by the lower overall torsional level and higher planarity than *cis*-PPV.^62^ We also calculated the transition dipole moment (μ) and oscillator strength (*f*) for all PPVs, and as expected, poly-*cis*-**1-***b*-*trans*-**2** had a higher transition dipole moment (Table 2). The *x*, *y*, and *z* vector components of the ground- to excited-state transition electric dipole moments are provided in Table 2 for μ. The strong dependence of the random TPA cross section on the transition dipole moment (eq 2) therefore explains why poly-*cis*-**1**-*b*-*trans*-**2** has a greater TPA cross-section value compared to poly-*cis*-**1**-*b*-*cis*-**2**.

**Table 2:** Calculated Transient Dipole Moment and Oscillator Strength for All-*cis* and All-*trans* PPV^a^

	poly-*cis*-**1**-*b*-*trans*-**2**		poly-*cis*-**1**-*b*-*cis*-**2**	
excited state	**μ**^*i→f*^**(a.u.)**	*f*^*i→f*^	**μ**^*i→f*^**(a.u.)**	*f*^*i→f*^
ES1	6.1, 0.1, 0.2	2.78	–1.7, 0.9, −0.2	0.35
ES2	–0.4, 1.2, −0.1	0.14	0.7, −2.2, −0.3	0.53
ES3	–0.7, −0.1, 0.3	0.05	–1.4, 0.4, −0.2	0.23

a The CAM-B3LYP-D3/6-311+G(d,p)-SMD(CHCl_3_) level of theory was used for the three lowest singlet excitations.

### Time-Resolved Fluorescence Upconversion

To have insight into early time relaxation dynamics, time-resolved fluorescence studies of poly-*cis*-**1**-*b*-*cis*-**2** and poly-*cis*-**1**-*b*-*trans*-**2** were performed at the emission wavelength of 517 nm upon excitation at 400 nm using the fluorescence upconversion technique (Figure 3D). As shown in Figure 3D, decay curves clearly show that there is a faster fluorescence decay for poly-*cis*-**1**-*b*-*trans*-**2** compared to poly-*cis*-**1**-*b*-*cis*-**2**. All the time profiles are triexponentially fit with a relatively fast rise component of ∼0.153–0.166 ps and predominant slow components in the ps time domain. All of the fitted decay parameters are given in Table 3. Both poly-*cis*-**1**-*b*-*cis*-**2** and poly-*cis*-**1**-*b*-*trans*-**2** show almost identical rise times. However, the fluorescence decay lifetimes for both PPVs are different. For poly-*cis*-**1**-*b*-*trans*-**2**, the shorter time constant becomes faster from 155 to 28.1 ps along with a rise of a very fast time constant [0.166 ps]. Again, the longer component changes from 604 to 413 ps indicates a decrease in radiative processes in the case of poly-*cis*-**1**-*b*-*trans*-**2** relative to poly-*cis*-**1**-*b*-*cis*-**2**. The decrease in radiative processes of the decay dynamics suggests that a more efficient charge transfer occurs in poly-*cis*-**1**-*b*-*trans*-**2**.

**Table 3:** Summary of the Fluorescence Upconversion with Both Copolymers^a^

**samples**	**τ**_**1**_**(ps) (a**_**1**_**%)**	**τ**_**2**_**(ps) (a**_**2**_**%)**	**τ**_**3**_**(ps) (a**_**3**_**%)**
poly-*cis*-**1-***b*-*cis*-**2**	0.153 ± 0.004	155 ± 1.22	604 ± 10.4
	(−100)	(63)	(37)
poly-*cis*-**1-***b*-*trans*-**2**	0.166 ± 0.002	28.1 ± 1.00	413 ± 0.66
	(−100)	(10)	(90)

a A negative amplitude indicates that the corresponding components are rise components.

### Femtosecond Transient Absorption

**Figure 4:**
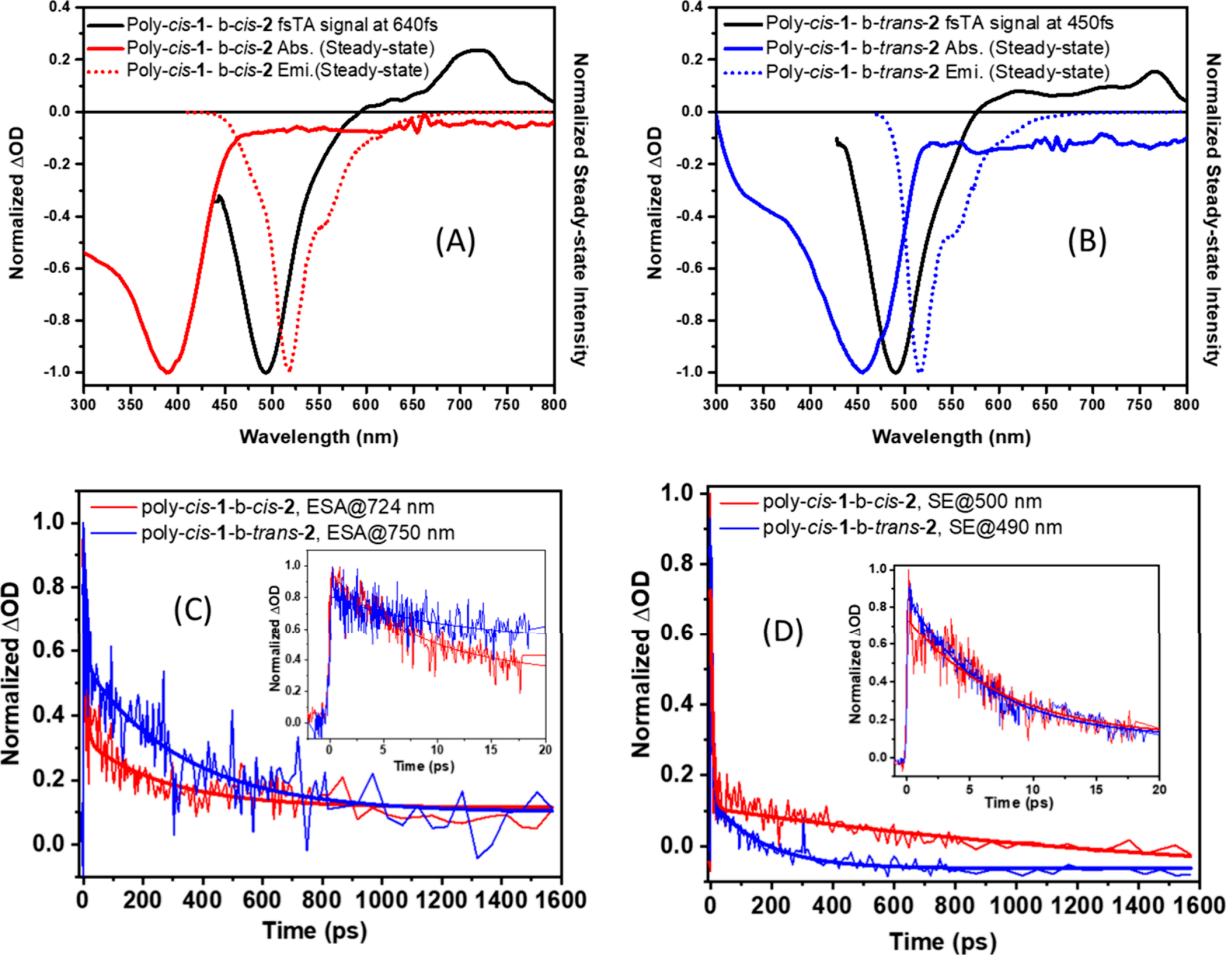
Femtosecond transient absorption spectra (*fs*TAS) for poly-*cis*-**1**-*b*-*cis*-**2** (A) and poly-*cis*-**1**-*b*-*trans*-**2** (B) corroborated with steady-state absorption and emission spectra. Time traces for excited-state absorption (C) and stimulated emission (D) of poly-*cis*-**1**-*b*-*cis*-**2** and poly-*cis*-**1**-*b*-*trans*-**2** using 400 nm as the excitation wavelength for both compounds. The insets in panels C and D show the time traces corresponding to a 20 ps time window.

To gain more insight into the charge carrier distribution in the excited state and their relaxation and recombination dynamics of both PPVs in a subpicosecond to picosecond time scale, we further performed time-resolved transient absorption (TA) experiments. Figure 4A,B (and Figure S8) presents the time-resolved TA spectra of the excited-state population following photoexcitation of the PPV samples in chloroform at room temperature. The pump source at 400 nm was used for both poly-*cis*-**1**-*b*-*cis*-**2** and poly-*cis*-**1**-*b*-*trans*-**2**, with probing of the entire visible region. The initial spectra consist of a stimulated emission (SE) around 500 nm (for poly-*cis*-**1**-*b*-*cis*-**2**) and 490 nm (for poly-*cis*-**1**-*b*-*trans*-**2**) that overlaps with ground-state bleach (GSB) for poly-*cis*-**1-***b*-*cis*-**2** and poly-*cis*-**1**-*b*-*trans*-**2**, respectively, and the broad excited-state absorption (ESA) band beyond 600 nm peaking at 724 and 750 nm for poly-*cis*-**1**-*b*-*cis*-**2** and poly-*cis*-**1**-*b*-*trans*-**2**, respectively. A blue shift (like the steady-state emission spectra) and spectral narrowing of the SE peak are observed for poly-*cis*-**1**-*b*-*trans*-**2** relative to poly-*cis*-**1**-*b*-*cis*-**2**. Figure 4C shows the ESA dynamics of poly-*cis*-**1**-*b*-*cis*-**2** and poly-*cis*-**1**-*b*-*trans*-**2** at 724 and 750 nm, respectively. Figure 4D shows the SE dynamics of poly-*cis*-**1**-*b*-*cis*-**2** and poly-*cis*-**1**-*b*-*trans*-**2** at 500 and 490 nm, respectively. The insets of Figure 4C,D represent the rise/decay dynamics in the 20 ps time domain, thus providing a better picture of the ultrafast time scale. All SE decays were well fitted by biexponential functions with characteristic lifetime values of 7.5 ps (73%) and >1600 ps (27%) (greater than the experimental time window) for poly-*cis*-**1**-*b*-*cis*-**2** and 5.8 ps (78.8%) and 167 ps (21.2%) for poly-*cis*-**1**-*b*-*trans*-**2**, respectively, which nicely corroborate with the fluorescence decay time measured by the fluorescence upconversion technique. The shortening of time signifies the faster excited-state energy/electron transfer, which eventually makes the bleach recovery faster in the case of poly-*cis*-**1**-*b*-*trans*-**2**. In addition, all ESA decays were also fitted biexponentially with lifetime values of 7.1 ps (67%) and 275 ps (33%) for poly-*cis*-**1**-*b*-*cis*-**2** at 724 nm and 8.3 ps (64%) and 332 ps (36%) for poly-*cis*-**1**-*b*-*trans*-**2** at 750 nm, respectively. Instead of ESA decay, we can also see a very fast rise component with lifetime values of 0.249 ps (for poly-*cis*-**1**-*b*-*cis*-**2**) and 0.273 ps (for poly-*cis*-**1**-*b*-*trans*-**2**), respectively.

The TA data measured for all-PPV are listed in Table 4. The early relaxation originates from the strong coupling between electronic and vibrational states. Hence, the fast kinetic processes are attributed to delocalized exciton states (sub ps) and vibrational cooling (few ps).^38^ Subsequently, the excited-state dynamics is dominated by intrachain charge/energy transfer on a characteristic time scale of tens of ps.^63,64^ This electronic charge/energy transfer occurs prior to emission, which generally stems from localized low-energy sites.^65−68^

**Table 4:** Transient Absorption Data for Poly-*cis*-**1**-*b*-*cis*-**2** and Poly-*cis*-**1**-*b*-*trans*-**2**^a^

**stimulated emission dynamics**		
**samples**	**t**_**1**_**(ps), (a**_**1**_**%)**	**t**_**2**_**(ps), (a**_**2**_**%)**
poly cis-**1**-*b*-cis-**2**	7.5 ± 0.38 (73)	>1600 ± 344 (27)
poly-*cis*-**1**-*b*-*trans*-**2**	5.8 ± 0.21 (78.8)	167 ± 22 (21.2)

**excited-state absorption dynamics**			
**samples**	**t**_**1**_**(ps), (a**_**1**_**%)**	**t**_**2**_**(ps), (a**_**2**_**%)**	**t**_**3**_**(ps), (a**_**3**_**%)**
poly-*cis*-**1**-*b*-*cis*-**2**	0.249 ± 0.09 (−100)	7.1 ± 0.56 (67)	275 ± 77 (33)
poly-*cis*-**1**-*b*-*trans*-**2**	0.273 ± 0.12 (−100)	8.3 ± 0.52 (64)	332 ± 64 (36)

a A negative amplitude indicates that the corresponding components are rise components.

In the case of SE dynamics, the *t*_1_ component [for poly-*cis*-**1**-*b*-*cis*-**2**, *t*_1_ = 7.5 ps, and for poly-*cis*-**1**-*b*-*trans*-**2**, *t*_2_ = 5.8 ps] is assigned to the vibrational cooling of the S_1_ state, whereas the *t*_2_ component [for poly-*cis*-**1**-*b*-*cis*-**2**, *t*_2_ = >1600 ps, and for poly-*cis*-**1**-*b*-*trans*-**2**, *t*_2_ = 167 ps] is assigned to the relaxation time from S_1_ to the ground state (S_0_). The shortening of the time constant signifies the more efficient excited-state energy/electron transfer that eventually makes the recombination faster in the case of poly-*cis*-**1**-*b*-*trans*-**2** because of its effective conjugation length, high dipole moment, and increased π–π stacking compared to poly-*cis*-**1**-*b*-*cis*-**2**.

The three excited-state components are characterized as hot S_1_ (hot singlet state), S_1_ (singlet state), and S_1_ to the ground state (S_0_). The hot S_1_ is short-lived (249 fs for poly-*cis*-**1**-*b*-*cis*-**2** and 273 fs for poly-*cis*-**1**-*b*-*trans*-**2**), which is attributed to the initial excitation to the hot S_1_ state of the PPVs. The hot S1 state decays very fast to the vibrationally relaxed S_1_ state whose lifetime is found to be 7.1 and 8.3 ps for poly-*cis*-**1**-*b*-*cis*-**2** and poly-*cis*-**1**-*b*-*trans*-**2**, respectively. Finally, it recombines to the ground state (S_0_) with the time constant of 275 and 332 ps for poly-*cis*-**1**-*b*-*cis*-**2** and poly-*cis*-**1**-*b*-*trans*-**2**, respectively. The time scale of the ES decay of S_1_–S_0_ is found to be very close to that of the upconversion decay trace. It is important to note that the excited-state decay for poly-*cis*-**1**-*b*-*trans*-**2** is relatively slower than that for poly-*cis*-**1**-*b*-*cis*-**2**, which is due to the efficient charge transfer nature of poly-*cis*-**1**-*b*-*trans*-**2** and generation of a long-lived charge transfer state that is beneficial for a light-harvesting system.

### Entangled Two-Photon Absorption

Entangled two-photon absorption, a nonlinear optical process, was utilized to acquire a better understanding of how quantum light (nonclassical) interacts with poly-*cis*-**1**-*b*-*cis*-**2** and poly-*cis*-**1**-*b*-*trans*-**2**. The mechanism for this transition involves the absorption of two entangled photons, which are quantum correlated with each other and induce unique photophysics during the ETPA transition. In classical TPA, the two photons are not correlated with each other, and so neither are the two absorption events because the two photons can be absorbed randomly. Hence, ETPA scales linearly with the input photon rate rather than quadratically for classical TPA. The linear trend yields an enhancement in the absorption rate at low input intensities compared to classical TPA, allowing two-photon transitions to be probed at extremely small photon rates where the molecule cannot be damaged. Thus, this study enhances the molecular sensitivity in probing the photophysical characteristics of poly-*cis*-**1**-*b*-*cis*-**2** and poly-*cis*-**1**-*b*-*trans*-**2** with extremely low photon flux preventing photobleaching and light-mediated toxicity.

**Figure 5:**
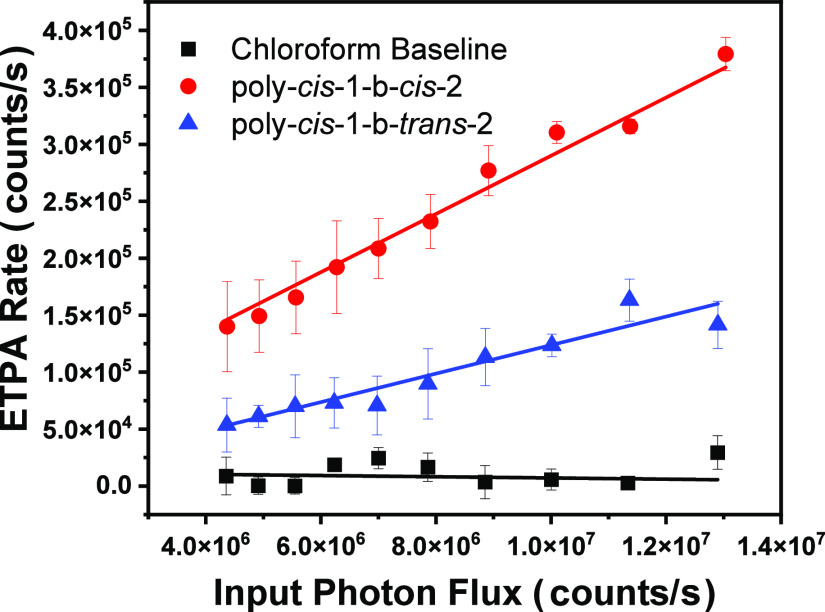
Entangled two-photon absorption as a function of input photon flux (i.e., excitation power) for poly-*cis*-**1**-*b*-*cis*-**2** and poly-*cis*-**1**-*b*-*trans*-**2** in chloroform solution. ETPA scales linearly with the input photon flux rather than quadratically for classical TPA. The linear trend yields an enhancement in the absorption rate at low input intensities compared to classical TPA. The ETPA rate is determined to be the difference between the photon counts per second transmitted through the solvent (chloroform) and the sample (PPVs) solution for a given input photon flux.

Figure 5 shows the ETPA rate as a function of the input photon flux for poly-*cis*-**1**-*b*-*cis*-**2** and poly-*cis*-**1**-*b*-*trans*-**2** in chloroform. Both poly-*cis*-**1**-*b*-*cis*-**2** and poly-*cis*-**1**-*b*-*trans*-**2** absorbed entangled light in magnitudes higher than the baseline, as expected. Furthermore, at relatively similar concentrations, poly-*cis*-**1**-*b*-*cis*-**2** (8.9 μM) exhibited an ETPA absorption higher than that of poly-*cis*-**1**-*b*-*trans*-**2** (8.4 μM). This finding implies that poly-*cis*-**1**-*b*-*cis*-**2** absorbs entangled photons better than poly-*cis*-**1**-*b*-*trans*-**2** in a chloroform solution. In general, the ETPA cross section, σ_e_, is given by^69^
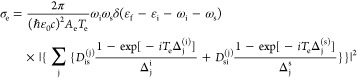
3where ℏ is Planck’s constant; ε_0_ is vacuum permittivity; *c* is the speed of light; *A*_e_ and *T*_e_ are entanglement area and entanglement time, respectively; ω_i_ and ω_s_ are the frequencies of the idler and signal photons; δ (ε_f_ – ε_i_ – ω_i_ – ω_s_) enforces energy conservation; and ε_i_ are the energy eigenvalues of the ground and excited states, respectively. *D*_is_^(*j*)^ = < Ψ_i_|*e*_i_μ|Ψ_*j*_ > < Ψ_*j*_|*e*_s_μ|Ψ_f_ > gives the transition dipole matrix elements, and Δ_*j*_^(*k*)^ = ε_j_ – ε_i_ – ω_*k*_ is the detuning energy where *k* = i or s, referring to the signal and idler photons. Like classical TPA, the ETPA cross section is also directly proportional to the transition dipole moment; thus, a similar trend in ETPA cross section would be expected. Interestingly, the ETPA cross section (Table 1) for poly-*cis*-**1**-*b*-*cis*-**2** (5.0 × 10^–18^ cm^2^/molecule) was found to be twice as large as that for poly-*cis*-**1**-*b*-*trans*-**2** (2.2 × 10^–18^ cm^2^/molecule), indicating that *cis*-PPV is more sensitive to entangled light, which can be useful for quantum sensing and quantum imaging applications.

**Figure 6:**
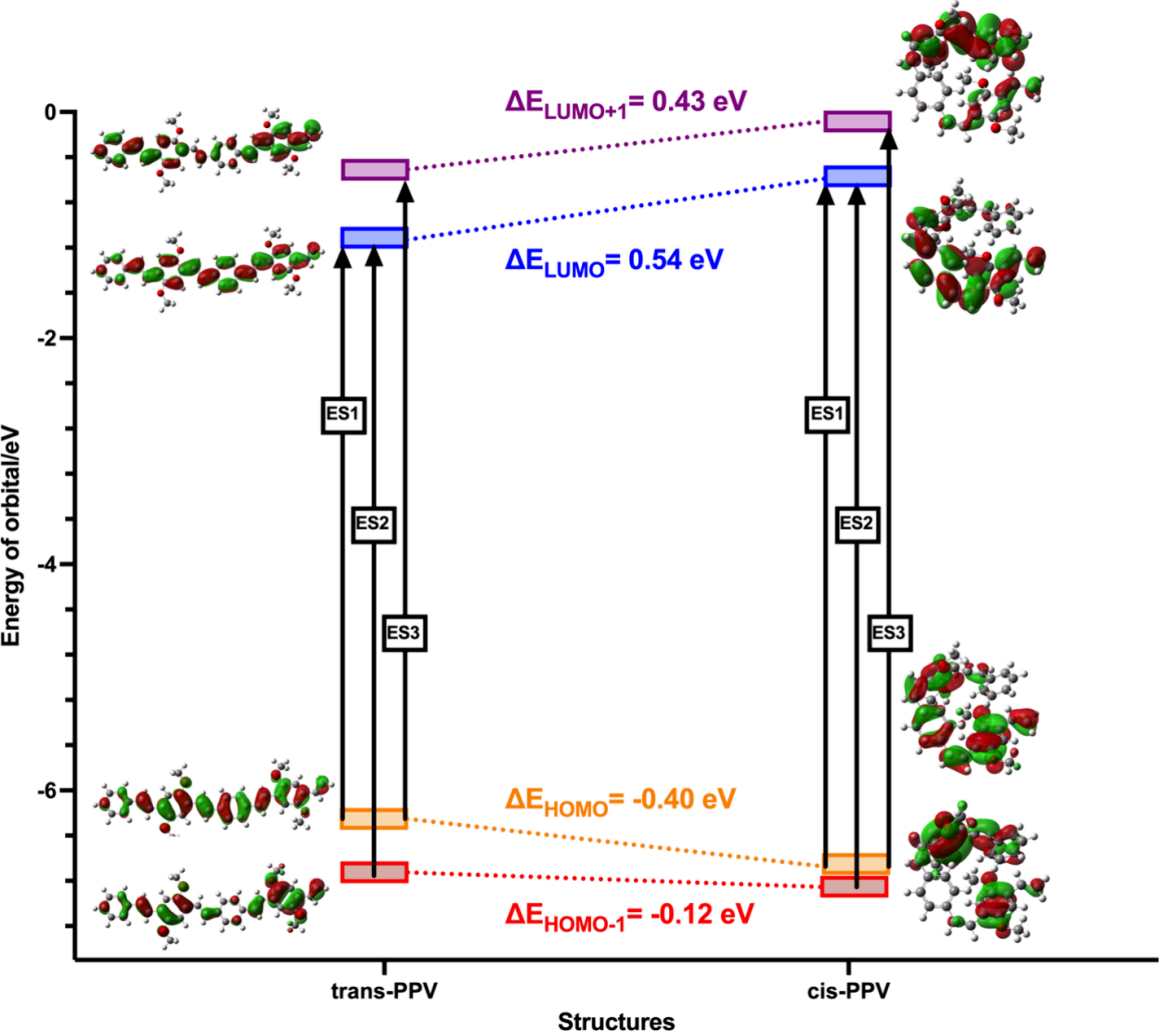
Frontier molecular HOMO – 1 through LUMO + 1 orbitals of *trans*- (left panel) and *cis*-PPV (right panel) calculated at the CAM-B3LYP-D3/6-311+G(d,p)-SMD(CHCl_3_) level of theory.

The capability of these PPVs to absorb entangled light may be the cause of this opposite trend. However, the ETPA cross section also depends on the electronic energy levels of the molecule. Generally, in each molecule, there are certain electronic energy states that are not easily accessible by classical light but may be accessible with entangled light. It has been described by Chouk et al. that *cis*-PPV has a higher energy band gap and lower HOMO level than *trans*-PPV.^58^ In our TD-DFT studies performed on *trans*- and *cis*-PPV, we observed a similar decrease in the HOMO energy level by −0.40 eV from *trans*- to *cis*-PPV (Figure 6 and Figure S15), respectively. The decrease in the HOMO orbital energy coupled with the increase in energy of the LUMO and LUMO + 1 orbitals increased the band gap of the *cis*-PPV. Interestingly, the HOMO – 1 orbital also decreased in energy level by −0.12 eV (Figure 6). Presumably, this lower-lying HOMO – 1 electronic energy state could enable *cis*-PPV to absorb a substantial quantity of entangled photons, leading to a greater ETPA cross section than *trans*-PPV. The TPA cross section is attributed to a virtual state involving an intermediate state that has been found to have a large ETPA cross section. However, materials with a large classical TPA cross section attributed to a dipole transition without the involvement of an intermediate state were found to be nearly transparent to an entangled photon source. According to the literature, these materials undergo TPA through a direct transition^60^ corresponding to the large change in permanent dipole moment between the ground and excited states, similar to our observation with poly-*cis*-**1**-*b*-*trans*-**2**. This mechanism can take place without the presence of virtual states close to the resonance with the photons. In the case of poly-*cis*-**1**-*b*-*cis*-**2**, the one-photon excitation in the two-photon excitation process is close to the resonance excitation wavelength, which might explain the small detuning energy that increased the ETPA cross section. However, in the case of poly-*cis*-**1**-*b*-*trans*-**2**, the one-photon excitation is relatively far from resonance, potentially resulting in a large detuning energy that decreases the ETPA cross section. To gain a better understanding as to why the *cis*-PPV showed a lower HOMO – 1 energy level, we sought to simulate the amount of orbital delocalization in the *trans*- vs *cis*-PPV systems by measurement of the orbital delocalization index (ODI) for HOMO and HOMO – 1 orbitals. Interestingly, although both HOMO and HOMO – 1 orbitals were lower in energy for the *cis*-PPV, less orbital delocalization was observed (Tables S8 and S9), contrary to what would be expected. As a result, single-point calculations at the CAM-B3LYP-D3/6-311G(d,p)-SMD(CHCl_3_) level of theory were performed on the individual styrene (1)/(3) and 1,4-dimethoxy-2-vinylbenzene (2)/(4) fragments of *cis*- (Figure S14) and *trans*-PPV (Figure S13). Plotting the HOMO – 1, HOMO, LUMO, and LUMO + 1 energies of each fragment, we determined that the HOMO and HOMO – 1 orbitals significantly decreased in energy from *trans*- to *cis*-PPV for the majority of fragments (Figure S15). The styrene fragment 1 (*trans*-PPV(1) vs *cis*-PPV(1)) saw the largest decrease in HOMO orbital energy from *trans*- to *cis*-PPV (Δ*E*_HOMO_(1) = −0.16 eV). The 1,4-dimethoxy-2-vinylbenzene fragment 2 (*trans*-PPV(2) vs *cis*-PPV(2)) saw the largest decrease in HOMO – 1 orbital energy from *trans*- to *cis*-PPV (Δ*E*_HOMO–1_(2) = −0.32 eV), leading to a decrease in the entire *cis*-PPV HOMO – 1 orbital energy (Table S10) modulated by the change in dihedral angle (Figure S17). These results suggest that the quantum interference between entangled photons and the interacting matter can provide changes to two-photon absorption signals that are not observed classically due to a lower-lying HOMO – 1 electronic state in *cis*-PPV.

## Conclusions

This comprehensive spectroscopic investigation of poly-*cis*-**1**-*b*-*cis*-**2** and poly-*cis*-**1**-*b*-*trans*-**2** demonstrates that the olefin geometry of the conjugated polymer significantly impacts the two-photon absorption properties. When TPA cross sections are compared, poly-*cis*-**1**-*b*-*trans*-**2** has a greater classical TPA cross section than poly-*cis*-**1**-*b*-*cis*-**2**, suggesting a more efficient ICT, which is in line with our femtosecond transient absorption studies where we found a relatively long-lived charge transfer state for poly-*cis*-**1**-*b*-*trans*-**2**. These findings might have strong implications for the potential uses of PPVs as fluorophores. Indeed, whereas all-*trans* PPV might be suitable for applications including the use of a number of different OLEDs, two-photon imaging, and photovoltaic applications because of a more efficient ICT, all-*cis* PPV appears to be more sensitive to entangled light (larger ETPA cross section), which is desirable for quantum sensing and quantum imaging applications. DFT calculations support our hypothesis that the all-*cis* PPV variant possesses a lower-lying HOMO – 1 state comparatively to the all-*trans* congener, which suggests a more energetically favorable interaction with entangled photons that could result in the larger ETPA cross section observed experimentally. We anticipate that the ability to tune both classical TPA and nonclassical ETPA cross sections through synthetic structural modification of the olefin geometry in PPVs will help inform the future design of chromophores for applications in this field.

## Supporting Information Available

Reagent, analytical information, monomer synthesis, diblock copolymer synthesis, size exclusive chromatography, quantum yield calculations, computational methods, femtosecond transient absorption spectra, and references (PDF)
