# Supplementary material for: Linear and Nonlinear Optical Properties of All-cis and All-trans Poly(p-phenylenevinylene)
Source: J Phys Chem C Nanomater Interfaces. 2024 Feb 2;128(6):2518–28. doi: 10.1021/acs.jpcc.3c07082 (PMC10875663; doi:10.1021/acs.jpcc.3c07082)
Supplement: Supplementary file 1 — jp3c07082_si_001.pdf [file jp3c07082_si_001.pdf]

Supporting Information:

Linear and Nonlinear Optical Properties of All-*cis* and All-*trans* Poly(*p*-phenylene vinylene)

Haraprasad Mandal,[1] *Olusayo J. Ogunyemi,*[2] *Jake L. Nicholson,*[3] *Meghan E. Orr,*[1] *Remy F. Lalisse,*[3] *Ángel Rentería-Gómez,*[3] *Achyut R. Gogoi,*[3] *Osvaldo Gutierrez,*[3] *Quentin Michaudel,*[3,4*] *Theodore Goodson, III* [1,2*]

[1] Department of Chemistry, University of Michigan, Ann Arbor, Michigan 48109, United States.

[2] Department of Macromolecular Science & Engineering, University of Michigan, Ann Arbor, Michigan 48109, United States.

[3] Department of Chemistry, Texas A&M University, College Station, Texas 77843, United States.

[4] Department of Materials Science and Engineering, Texas A&M University, College Station, Texas, 77843, United States

Experimental Procedures

General Reagent Information S4

General Analytical Information S4

Monomer Synthesis S4

Scheme S1. Synthesis of monomer **2** S4

Diblock Copolymer Synthesis S6

Figure S1. ^{1}H NMR (400 MHz, $CDCl_3$) spectrum of poly-*cis*-**1**-*b*-*cis*-**2** S6

Figure S2. ^{13}C NMR (125 MHz, $CDCl_3$) spectrum of poly-*cis*-**1**-*b*-*cis*-**2** S7

Figure S3. ^{1}H NMR (400 MHz, $CDCl_3$) spectrum of poly-*cis*-**1**-*b*-*trans*-**2** S8

Figure S4. ^{13}C NMR (125 MHz, $CDCl_3$) Spectrum of poly-*cis*-**1**-*b*-*trans*-**2** S8

Size Exclusion Chromatography S9

Table S1. Molecular weight and dispersity values S9

Figure S5. Size exclusion chromatography traces S9

Quantum Yield Calculations S9

Equation S1 .... S9

Figure S6. Integrated emission intensity vs. absorbance for poly-*cis*-**1**-*b*-*cis*-**2** and Coumarin 153 standard S10

Figure S7. Integrated emission intensity vs. absorbance for poly-*cis*-**1**-*b*-*trans*-**2** and Fluorescein standard S10

Femtosecond Transient Absorption Spectra S11

Figure S8. Femtosecond transient absorption spectra for poly-*cis*-**1**-*b*-*cis*-**2** and poly-*cis*-**1**-*b*-*trans*-**2** S11

Computational analysis of *trans*- and *cis*-PPV S11

Figure S9. Boltzmann weighted UV-Vis spectra of (a) *trans*- and (b) *cis*-PPV calculated at the CAM-B3LYP/6-311+G(d,p) level of theory in chloroform (SMD) S15

Figure S10. Boltzmann weighted UV-Vis spectra of (a) *trans*- and (b) *cis*-PPVcalculated at the B3LYP/6-311+G(d,p) level of theory in chloroform (SMD) S15

Figure S11. Boltzmann weighted UV-Vis spectra of (a) *trans*- and (b) *cis*-PPV calculated at the M06-2X/6-311+G(d,p) level of theory in chloroform (SMD) S16

Fragmentation model S16

Figure S12. Single point calculations for the HOMO and HOMO-1 orbitals

for the *trans*- (left panel) and *cis*-PPV (right panel) calculated at the CAM-B3LYP-D3/6-311G(d,p)-SMD($CHCl_3$) level of theory S17

Figure S13. Single point calculations for the HOMO and HOMO-1 orbitals for the *trans*-PPV fragments calculated at the CAM-B3LYP-D3/6-311G(d,p)-SMD($CHCl_3$) level of theory S18

Figure S14. Single point calculations for the HOMO and HOMO-1 orbitals for the *cis*-PPV fragments calculated at the CAM-B3LYP-D3/6-311G(d,p)-SMD ($CHCl_3$) level of theory S19

Figure S15. Comparison of the HOMO and HOMO-1 orbitals for the *trans/cis*-PPV fragments calculated at the CAM-B3LYP-D3/6-311G(d,p)-SMD ($CHCl_3$) level of theory S20

Figure S16. Plot of the HOMO and HOMO-1 orbitals with respect to the dihedral angle of fragment 1 where the blue line represents the dihedral angle of *cis*-PPV and the purple line represents the dihedral angle of *trans*-PPV calculated at the CAM-B3LYP-D3/6-311G(d,p)-SMD($CHCl_3$) level of theory....................................S21

Figure S17. Plot of the HOMO and HOMO-1 orbitals with respect to the dihedral angle of fragment 2 where the blue line represents the dihedral angle of *cis*-PPV and the purple line represents the dihedral angle of *trans*-PPV calculated at the CAM-B3LYP-D3/6-311G(d,p)-SMD($CHCl_3$) level of theory....................................S22

Figure S18. Plot of the HOMO and HOMO-1 orbitals with respect to the dihedral angle of fragment 3 where the blue line represents the dihedral angle of *cis*-PPV and the purple line represents the dihedral angle of *trans*-PPV calculated at the CAM-B3LYP-D3/6-311G(d,p)-SMD($CHCl_3$) level of theory....................................S23

Figure S19. Plot of the HOMO and HOMO-1 orbitals with respect to the dihedral angle of fragment 4 where the blue line represents the dihedral angle of *cis*-PPV and the purple line represents the dihedral angle of *trans*-PPV calculated at the CAM-B3LYP-D3/6-311G(d,p)-SMD($CHCl_3$) level of theory....................................S24

Steady-State Absorption and Emission....................................S25

Two-Photon Absorption (TPA) S25

Time-Resolved Fluorescence Up-Conversion....................................S25

Femtosecond Transient Absorption S26

References....................................S28

General Reagent Information: All reactions were carried out under an inert nitrogen atmosphere with dry solvents under anhydrous conditions unless otherwise stated. Dry dichloromethane (DCM), diethyl ether (Et_2O), tetrahydrofuran (THF), and toluene (PhMe) were obtained by passing the previously degassed solvents through activated alumina columns. Synthesis of polymers using stereoretentive ruthenium catalyst was carried out in a nitrogen-filled glove box (SG1800/750TS-F, VIGOR). Reagents were purchased at the highest commercial quality and used without further purification, unless otherwise stated. Yields refer to chromatographically and spectroscopically (^1H NMR) homogeneous material, unless otherwise stated. Reactions were monitored by thin layer chromatography (TLC) carried out on 250 µm SiliCycle SiliaPlate™ silica plates (F254), using UV light as the visualizing agent. Flash silica gel chromatography was performed using SiliCycle SiliaFlash® Irregular Silica Gel (60 Å, particle size 40–63 µm). The polymer was isolated after precipitation using an Eppendorf 5804 centrifuge.

General Analytical Information: The polymer sample was analyzed using a Tosoh EcoSec HLC 8320GPC system with a TSKgel SuperHM-M column and a TSKgel SuperH-RC column at a flow rate of 0.40 mL/min at 40 °C. THF stabilized with BHT was used as the eluent and the number-average molecular weight (M_n), weight-average molecular weight (M_w), and dispersity ($Đ$) for the polymer were calculated from a UV chromatogram against a TSKgel polystyrene standard. NMR spectra were recorded on Bruker Avance Neo 400 and Bruker Avance 500 instruments and were calibrated using residual undeuterated solvent as an internal reference ($CHCl_3$ @ 7.26 ppm ^1H NMR, 77.16 ppm ^{13}C NMR). The following abbreviations were used to explain NMR peak multiplicities: s = singlet, d = doublet, t = triplet, q = quartet, m = multiplet, br = broad.

Monomer Synthesis:

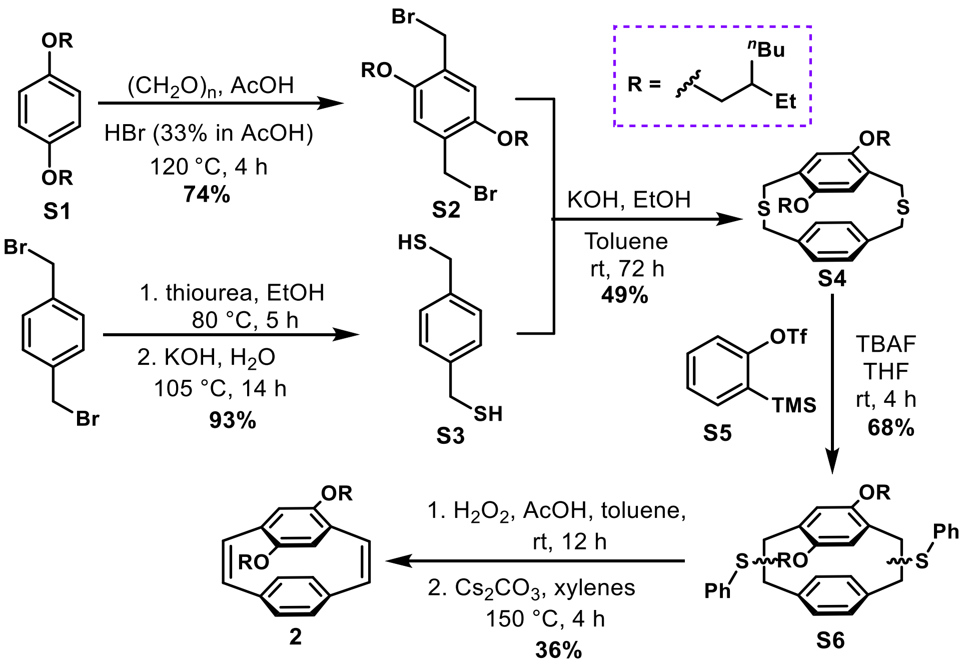

Scheme S1. Synthesis of monomer **2**.

N-^{n}Hexyl *exo*-norbornene diimide (1). Compound 1 was synthesized following literature procedure reported by Michaudel and coworkers and the spectroscopic data for this compound were identical to those reported in the literature.[1] ^{1}H NMR (500 MHz, $CDCl_3$) δ 6.28 (s, 2 H), 3.45 (t, *J* = 7.6 Hz, 2 H), 3.27 (s, 2 H), 2.66 (s, 2 H), 1.59–1.47 (m, 3 H), 1.33–1.21 (m, 7 H), 0.86 (t, *J* = 6.7 Hz, 3 H).

1,4-Di(2-ethylhexyloxy)benzene (S1). Compound **S1** was synthesized following literature procedure reported by Lozano and co-workers and the spectroscopic data for this compound were identical to those reported in the literature.[2] ^{1}H NMR (400 MHz, $CDCl_3$) δ 6.82 (s, 4 H), 3.79 (d, *J* = 5.8 Hz, 2 H), 3.78 (d, *J* = 6.1 Hz, 2 H) 1.75–1.62 (m, 2 H), 1.54–1.24 (m, 16 H), 0.93–0.87 (t, 12 H) ppm.

1,4-Bis(bromomethyl)-2,5-di(2-ethylhexyloxy)benzene (S2). Synthesis of compound **S2** was adapted from literature procedure reported by Lozano and coworkers.[2] **S1** (10.0 g, 30.0 mmol, 1.0 equiv) and paraformaldehyde (3.6 g, 120 mmol, 4.0 equiv) were added to a 500 mL round bottom flask, then a solution of HBr (33 wt% in acetic acid, 20 mL) diluted in an additional 140 mL of acetic acid was added slowly. A condenser was adapted onto the flask and the reaction mixture was stirred at 120 °C for 4 h. Once cooled to room temperature, the mixture was poured into water (200 mL) at 0 °C. The precipitated solid was filtered and washed with water several times. The solid residues were then washed with MeOH (3 × 20 mL), and compound **S2** was isolated as an off-white solid (11.6 g, 22.3 mmol, 74%). The spectroscopic data for this compound were identical to those reported in the literature.[2] ^{1}H NMR (400 MHz, $CDCl_3$) δ 6.84 (s, 2H), 4.51 (s, 4H), 3.87 (d, *J* = 5 Hz, 4H), 1.75–1.62 (m, 2H), 1.54–1.24 (m, 16H), 0.93–0.87 (t, 12H) ppm.

1,4-Bis(thiolatomethyl)benzene (S3). Compound **S3** was synthesized following literature procedure reported by Turner and co-workers and the spectroscopic data for this compound were identical to those reported in the literature.[3] ^{1}H NMR (400 MHz, $CDCl_3$) δ 7.28 (s, 4 H), 3.73 (d, *J* = 7.5 Hz, 4 H), 1.75 (t, *J* = 7.5 Hz, 2 H) ppm.

5,8-Di(2-ethylhexyloxy)-2,11-dithia[3,3]paracyclophane (S4). Compound **S4** was synthesized following literature procedure reported by Turner and co-workers and the spectroscopic data for this compound were identical to those reported in the literature.[4] ^{1}H NMR (400 MHz, $CDCl_3$) δ 6.96 (d, *J* = 8 Hz, 2 H), 6.89 (d, *J* = 8 Hz, 2 H), 6.42 (s, 2 H), 4.26 (d, *J* = 15 Hz, 2 H), 3.89–3.83 (m, 2 H), 3.82 (d, *J* = 15 Hz, 2 H), 3.76 (d, *J* = 15 Hz, 2 H), 3.68–3.62 (m, 2 H), 3.36 (d, *J* = 15 Hz, 2 H), 1.79–1.72 (m, 2 H), 1.68–1.33 (m, 16 H), 1.06–0.92 (m, 12 H) ppm.

2-(trimethylsilyl)phenyl trifluoromethanesulfonate (S5). Benzyne precursor **S5** was prepared according to literature procedure reported by Turner and co-workers and the spectroscopic data for this compound were identical to those reported in the literature.[3] ^{1}H NMR (400 MHz, $CDCl_3$) δ 7.54 (dd, *J* = 7.5, 1.9 Hz, 1 H), 7.44 (ddd, *J* = 8.3, 7.3, 1.9 Hz, 1 H), 7.38–7.31 (m, 2 H), 0.37 (s, 9 H) ppm.

Dithiaparacyclophane Isomers (S6). Compound **S6** was synthesized following literature procedure reported by Turner and co-workers and the spectroscopic data for this compound were identical to those reported in the literature.[4]

4,7-Di(2-ethylhexyloxy)-[2.2]paracyclophane-1,9-diene (2). Compound **2** was synthesized following literature procedure reported by Turner and co-workers and the spectroscopic data

for this compound were identical to those reported in the literature.[4] ^{1}H NMR (400 MHz, $CDCl_3$) δ 7.10 (d, J = 10 Hz, 2 H), 6.90 (d, J = 10 Hz, 2 H), 6.80 (d, J = 8 Hz, 2 H), 6.48 (d, J = 8 Hz, 2 H), 5.77 (s, 2 H), 3.72–3.65 (m, 2 H), 3.65–3.58 (m, 2 H), 1.77–1.59 (m, 2 H), 1.59–1.25 (m, 16 H), 1.04–0.79 (m, 12 H) ppm.

Diblock Copolymer Synthesis:

Poly-*cis*-**1**-*b*-*cis*-**2**:

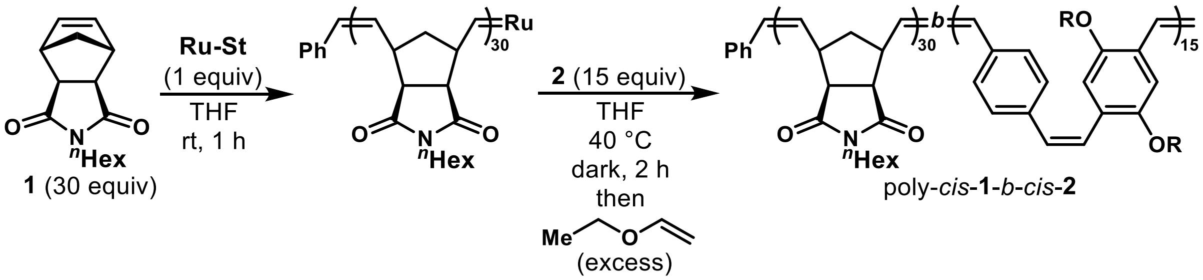

A solution of ruthenium catalyst **Ru-St** (8.6 mg, 9.9 µmol, 1 equiv) in deoxygenated THF (0.10 mL) was added to a reaction vial containing monomer **1** (73.3 mg, 296.4 µmol, 30 equiv) and a stir bar inside a nitrogen filled glove box. The reaction was stirred inside the glove box for 1 h at room temperature, and then 0.08 mL of the mixture was transferred to a second reaction vial containing monomer **2** (52.2 mg, 113.3 µmol, 15 equiv) and a stir bar. The mixture was stirred for 2 h at 40 °C in the dark inside the glovebox. The reaction was allowed to cool to room temperature, and then quenched with excess ethyl vinyl ether (0.1 mL) and left for 30 min at room temperature. The polymer was isolated by performing two cycles of precipitation with addition of methanol, centrifugation, and decantation. The isolated polymer was wrapped in aluminium foil and concentrated under reduced pressure before being stored under an inert atmosphere at –20 °C to prevent any undesired photoisomerization. ^{1}H NMR (400 MHz, $CDCl_3$) δ 7.21 (s, 4 H), 6.75 (s, 2 H), 6.64 (d, J = 12.3 Hz, 2 H), 6.49 (d, J = 12.3 Hz, 2 H), δ 5.60-5.37 (m, 4 H), 3.51–2.87 (m, 16 H), 2.37–1.95 (m, 2 H), 1.56–1.46 (m, 6 H), 1.41–1.13 (m, 30 H), 0.90–0.77 (m, 18 H) ppm. ^{13}C NMR (125 MHz, $CDCl_3$) δ 178.6, 178.4, 178.2, 150.5, 136.5, 133.5, 129.4, 128.9, 126.2, 125.5, 114.0, 71.4, 52.6, 52.4, 41.9, 41.6, 39.4, 39.0, 38.9, 31.5, 30.6, 29.1, 27.8, 26.7, 26.6, 24.0, 23.2, 22.7, 14.2, 14.1, 11.1 ppm.

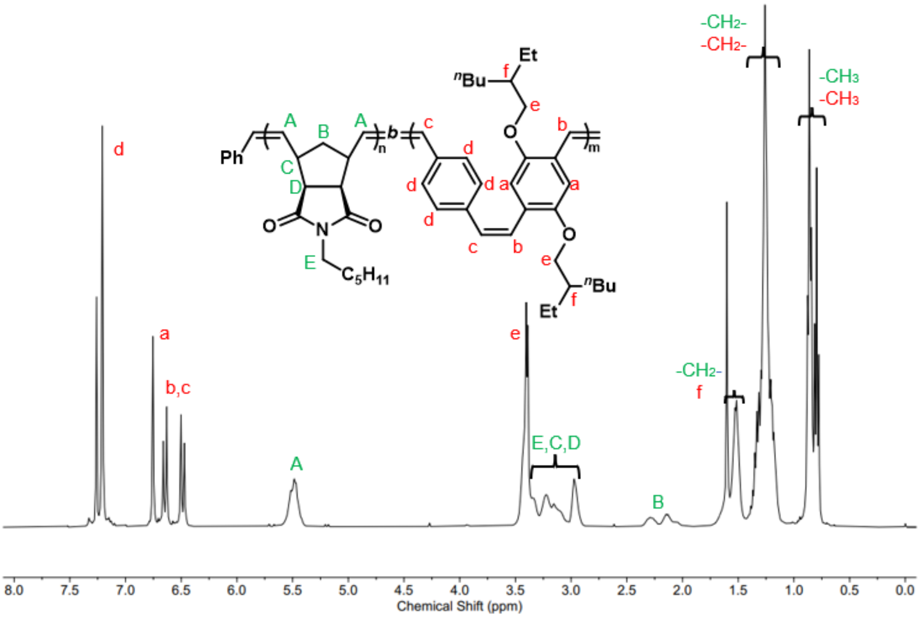

Figure S1. ^{1}H NMR (400 MHz, $CDCl_3$) spectrum of poly-*cis*-**1**-*b*-*cis*-**2**.

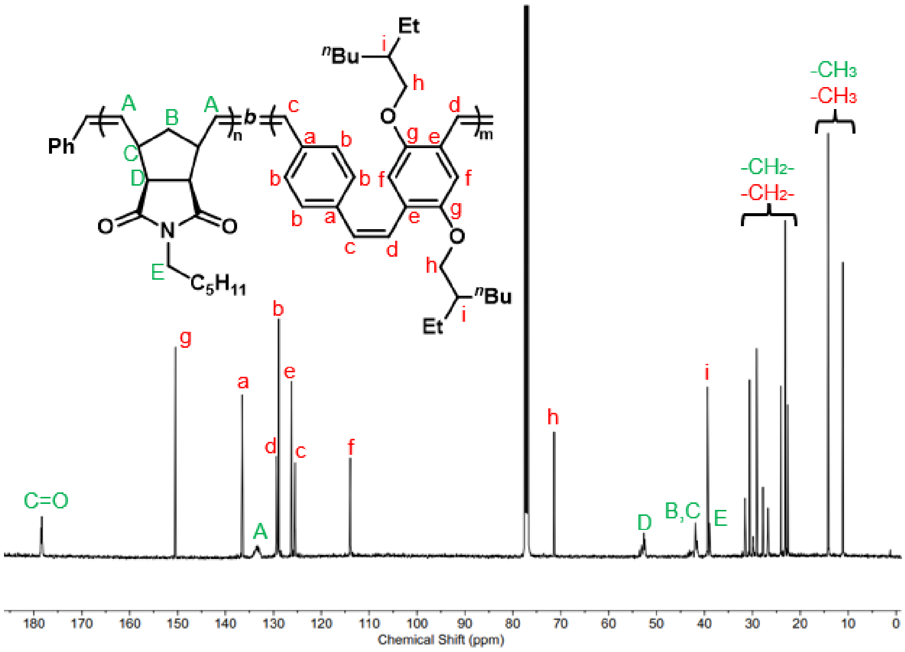

Figure S2. ^{13}C NMR (125 MHz, $CDCl_3$) spectrum of poly-*cis*-**1**-*b*-*cis*-**2**.

Poly-*cis*-**1**-*b*-*trans*-**2**:

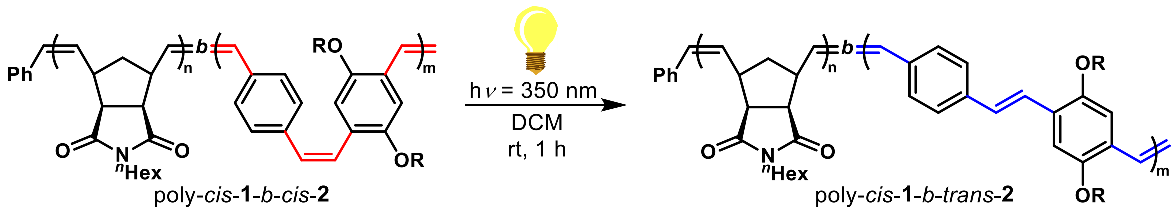

A solution (23 mg in 23 mL, C = 1 mg/mL) of poly-*cis*-**1**-*b*-*cis*-**2** was irradiated in DCM using two 350 nm UV lamps placed 1 cm away from the sample for 1 hour at room temperature to give poly-*cis*-**1**-*b*-*trans*-**2**. The sample was wrapped in aluminium foil and concentrated under reduced pressure before being stored under an inert atmosphere at –20 °C. ^{1}H NMR (400 MHz, $CDCl_3$) δ 7.65–7.30 (m, 6 H), 7.22–6.80 (m, 4 H), δ 5.68–5.26 (m, 4 H), 4.05–3.73 (m, 4 H), 3.51–2.87 (m, 12 H), 2.37–1.95 (m, 2 H), 1.93–1.79 (m, 2 H), 1.71–1.20 (m, 34 H), 1.04–0.80 (m, 18 H) ppm. ^{13}C NMR (125 MHz, $CDCl_3$) δ 178.6, 178.4, 178.2, 151.5, 137.4, 133.5, 128.6, 127.1, 127.0, 123.5, 110.5, 72.1, 52.6, 52.4, 41.9, 41.6, 40.0, 39.0, 38.9, 31.5, 31.2, 29.5, 27.8, 26.7, 26.6, 24.4, 23.2, 22.7, 14.3, 14.1, 11.5 ppm.

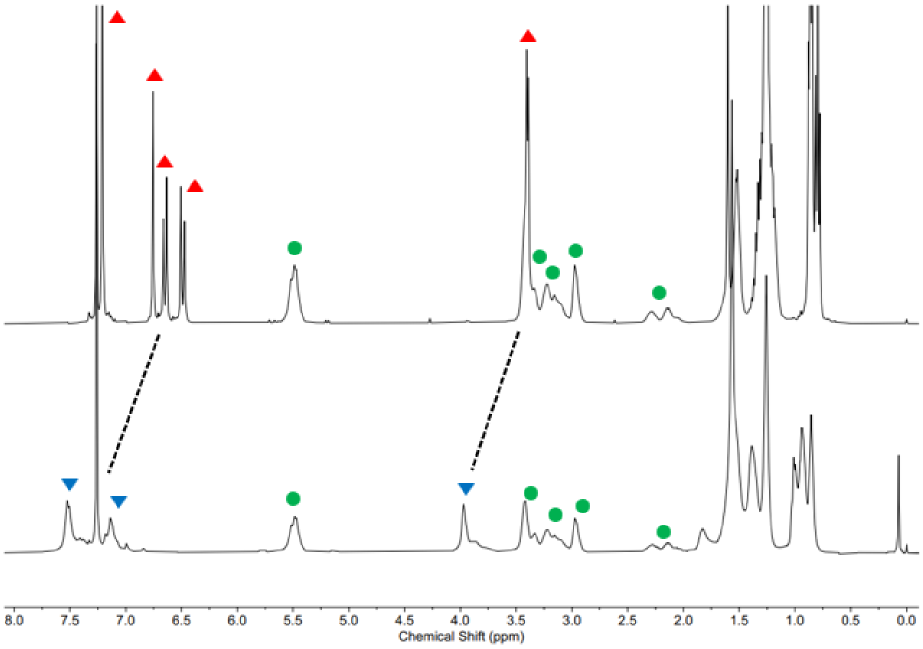

Figure S3. ^{1}H NMR (400 MHz, $CDCl_3$) spectra showing change in chemical shifts after photoisomerization of poly-*cis*-**1**-*b*-*cis*-**2** (red/green) to give poly-*cis*-**1**-*b*-*trans*-**2** (blue/green).

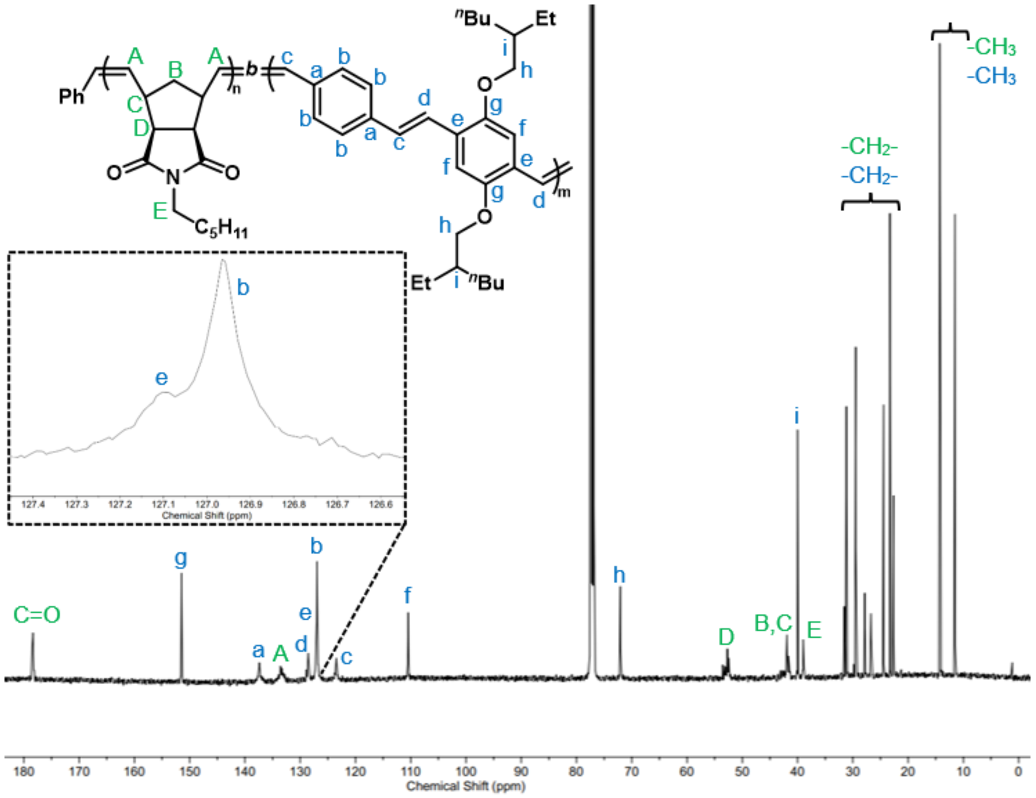

Figure S4. ^{13}C NMR (125 MHz, $CDCl_3$) spectrum of poly-*cis*-**1**-*b*-*trans*-**2**.

Size Exclusion Chromatography:

Table S1. Molecular weight and dispersity values for poly-*cis*-**1**-*b*-*cis*-**2** and poly-*cis*-**1**-*b*-*trans*-**2**.

Block copolymer	Target n,m	M_n^{theor} (kg/mol)	M_n^{exp} (kg/mol)	*Đ*
poly-*cis*-**1**-*b*-*cis*-**2**	30,15	14.5	16.3	1.20
poly-*cis*-**1**-*b*-*trans*-**2**	30,15	14.5	15.6	1.67

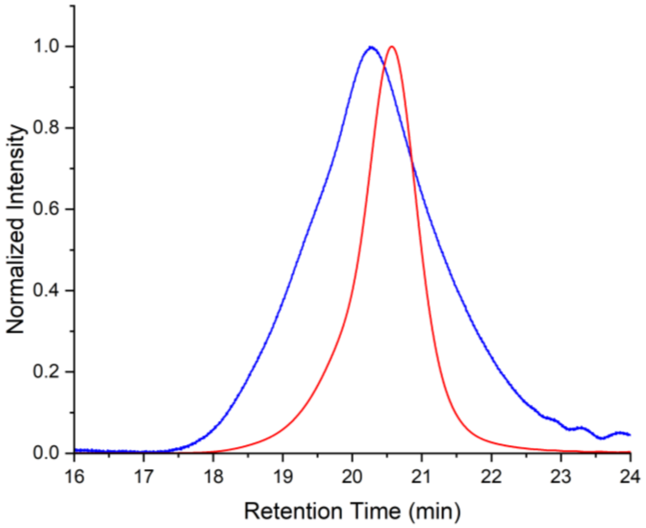

Figure S5. Size exclusion chromatography traces of poly-*cis*-**1**-*b*-*cis*-**2** (red) and poly-*cis*-**1**-*b*-*trans*-**2** (blue).

Quantum Yield Calculations:

Fluorescence quantum yields were measured in dilute solutions of poly-*cis*-**1**-*b*-*cis*-**2** and poly-*cis*-**1**-*b*-*trans*-**2** in chloroform (η = 1.45) against either Coumarin 153 in ethanol (Φ_{fl} = 0.53, η = 1.36) for poly-*cis*-**1**-*b*-*cis*-**2** or fluorescein in aq. NaOH (C = 0.1 M, Φ_{fl} = 0.89 , η = 1.33) for poly-*cis*-**1**-*b*-*trans*-**2**. Absorbance measurements were kept below 0.04 to reduce possible reabsorption effects. Fluorescence quantum yields were calculated based on the gradients (slope) of the plot of integrated emission versus absorbance at λ_{ex} using **Equation S1**.[5]

$$\Phi_{sample} = \Phi_{standard} \text{ x } \left(\frac{\text{Gradient}_{sample}}{\text{Gradient}_{standard}}\right) \text{ x } \left(\frac{\eta_{sample}}{\eta_{standard}}\right)^2 \qquad \textbf{(Equation S1)}$$

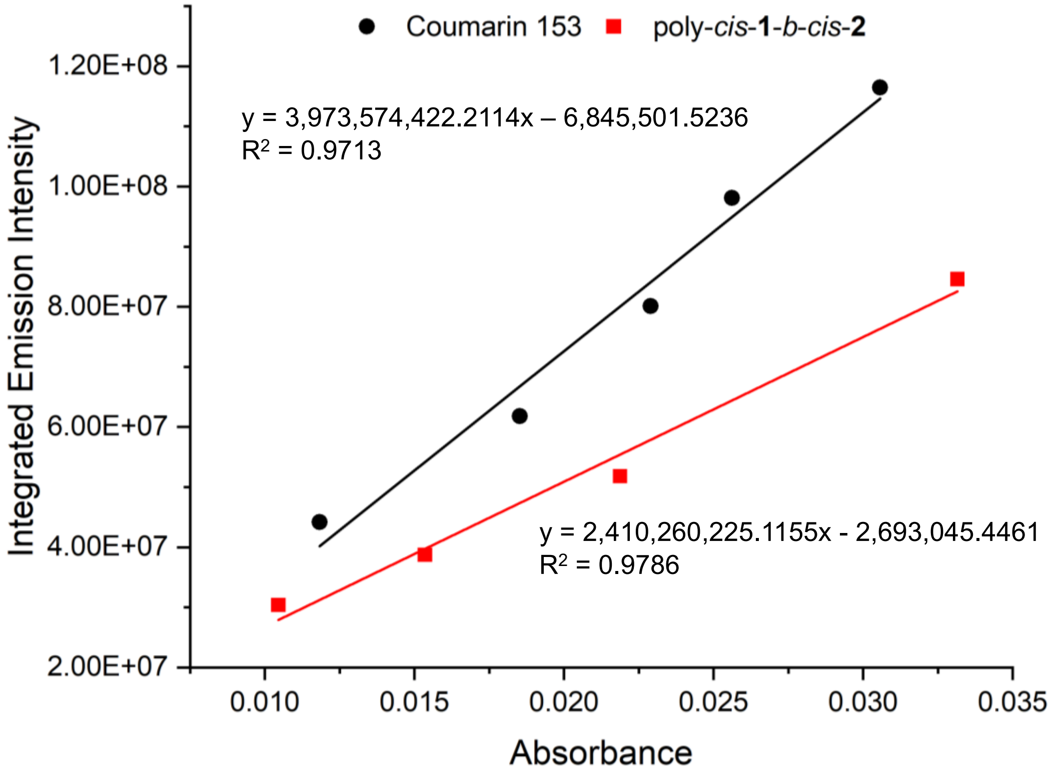

Figure S6. Plot of integrated emission intensity vs. absorbance for poly-*cis*-**1**-*b*-*cis*-**2** and Coumarin 153 (λ_{ex} = 389 nm).

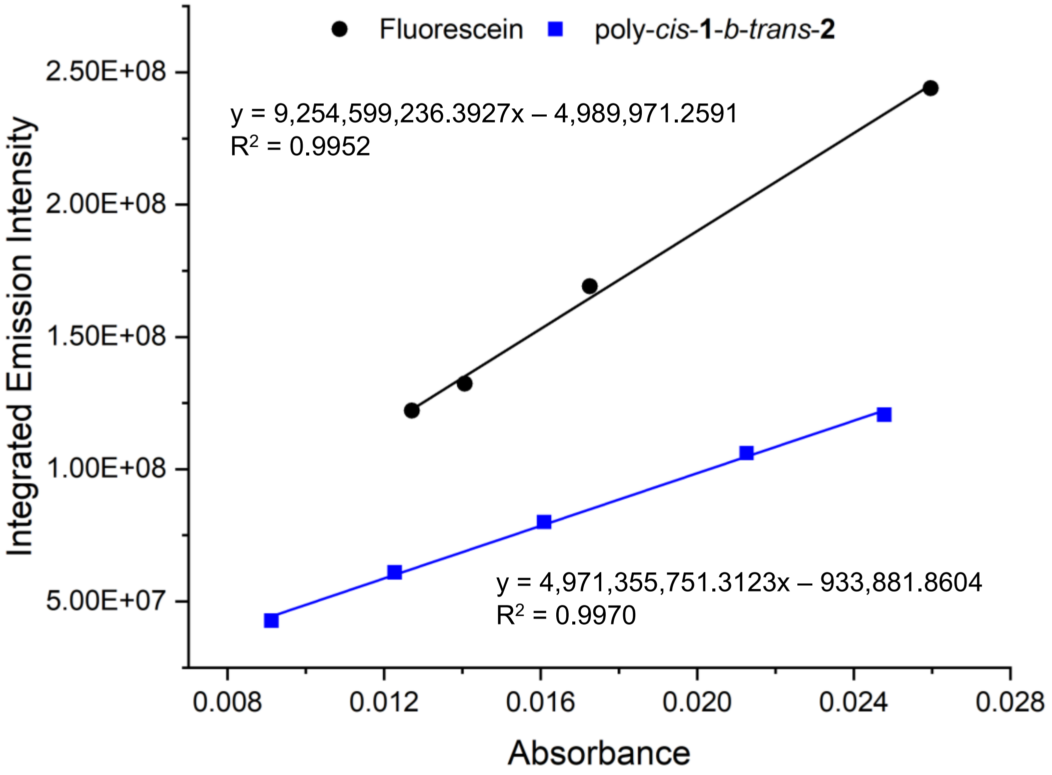

Figure S7. Plot of integrated emission intensity vs. absorbance for poly-*cis*-**1**-*b*-*trans*-**2** and Fluorescein (λ_{ex} = 442 nm).

Femtosecond Transient Absorption Spectra:

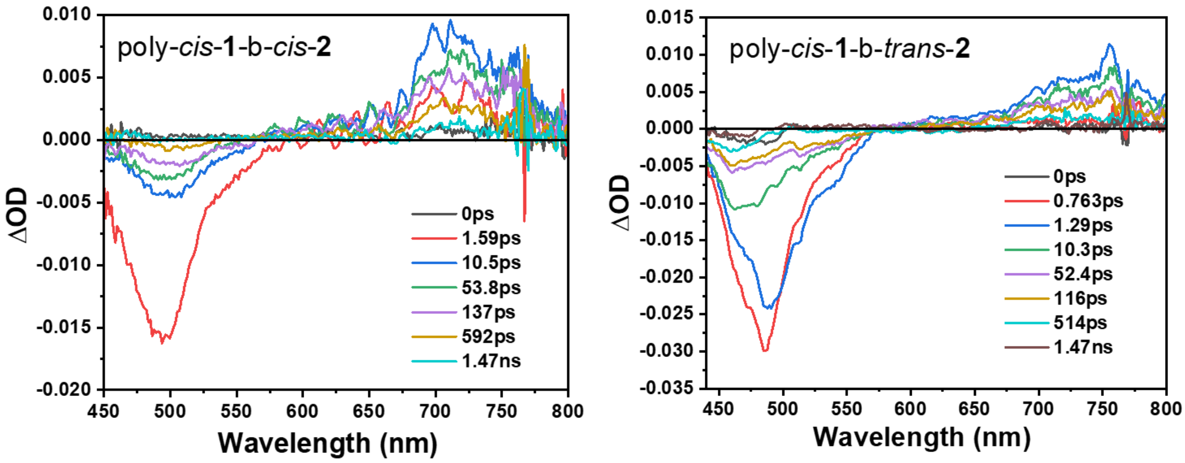

Figure S8. Femtosecond transient absorption spectra (*fs*TAS) for poly-*cis*-**1**-*b*-*cis*-**2** (A) and poly-*cis*-**1**-*b*-*trans*-**2** (B) at different delay times in chloroform.

Computational analysis of *trans*- and *cis*-PPV

The *trans*- and *cis*-PPV's were optimized using a two-step protocol of a conformational search with the OPLS3e[6] forcefield followed by a higher-level density functional theory (DFT) optimization at the CAM-B3LYP-D3/6-311+G(d,p)-SMD($CHCl_3$) level of theory.[7] For each system, a suggested structure was constructed, and an exhaustive conformational analysis was performed to generate 20 different starting conformations with the OPLS3e force field. using the Macromodel package of Schrodinger 23.[8] The 20 conformations were subjected to ground-state geometry optimizations carried out with the CAM-B3LYP density functional with the 6-311+G(d,p) basis set and GD3 empirical dispersion. Solvent effects were simulated in chloroform with the solvation model density method (SMD). All ground-state geometry optimizations were performed with Gaussian 16.[9] The stationary points from these efforts were then carried on to the subsequent UV−Vis studies performed at the same CAM-B3LYP-D3/6-311+G(d,p)-SMD($CHCl_3$) level of theory reported in the main manuscript. Higher-level optimizations and UV-Vis calculations were repeated with the B3LYP and M06-2X[10] functionals for comparison. Boltzmann weighted UV-Vis plots using the relative free energy (Tables S2 – S7) for *trans*- and *cis*-PPV were generated at the three different levels of theory (Figure S9 – S11).

At all levels of theory we found very similar observations in the TD-DFT simulations (Figure S9 – S11). For *trans*-PPV, one distinct UV-Vis excitation was observed at 413 (CAM-B3LYP-D3), 531 (B3LYP-D3), and 412 nm (M06-2X-D3) corresponding to one HOMO – LUMO singlet excitation (ES1, Figure S9(a) – S11(a)). The UV-Vis absorption peaks for *cis*-PPV were blue-shifted at 309 (CAM-B3LYP-D3), 369 (B3LYP-D3), and 311 nm (M06-2X-D3) when compared to *trans*-PPV (Figure S9(b) – S11(b)) correlating with experimental findings. At each level of theory, three distinct UV-Vis excitations were observed ES1 (HOMO – LUMO), ES2 (HOMO-1 – LUMO), and ES3 (HOMO – LUMO+1) illustrated in Figure 7.

Table S2. Energetic data for the top 20 conformations of *trans*-PPV calculated at the CAM-B3LYP-D3/6-311+G(d,p) level of theory in chloroform (SMD).

Conformation	Relative enthalpy at zero temperature (kcal/mol)	Relative enthalpy at 298 K (kcal/mol)	Relative free energy at 298 K (kcal/mol)
1	0.1	0.1	1.5
2	0.3	0.3	1.9
3	0.2	0.2	1.6
4	0.3	0.3	2.0
5	0.3	0.2	2.0
6	0.1	0.1	1.2
7	0.0	0.0	1.0
8	0.1	0.2	1.0
9	0.1	0.2	1.1
10	0.1	0.1	1.1
11	0.1	0.1	1.5
12	0.2	0.1	1.7
13	0.1	0.1	1.4
14	0.0	0.0	1.3
15	0.0	0.1	0.0
16	0.4	0.2	2.4
17	0.1	0.1	1.3
18	0.4	0.2	2.2
19	0.3	0.2	1.7
20	0.3	0.2	2.2

Table S3. Energetic data for the top 20 conformations of *trans*-PPV calculated at the B3LYP-D3/6-311+G(d,p) level of theory in chloroform (SMD).

Conformation	Relative enthalpy at zero temperature (kcal/mol)	Relative enthalpy at 298 K (kcal/mol)	Relative free energy at 298 K (kcal/mol)
1	0.2	0.1	0.7
2	0.1	0.1	0.6
3	0.1	0.1	1.0
4	0.1	0.1	0.7
5	0.0	0.0	0.1
6	0.1	0.2	0.2
7	0.1	0.1	0.7
8	0.1	0.1	0.2
9	0.1	0.1	0.3
10	REPEAT OF 3	REPEAT OF 3	REPEAT OF 3
11	0.3	0.3	0.7
12	0.0	0.0	0.0
13	0.0	0.0	0.4
14	0.2	0.2	0.3
15	0.0	0.0	0.3
16	0.1	0.1	0.5
17	0.1	0.0	0.6

18	0.4	0.3	1.6
19	0.4	0.2	1.4
20	0.3	0.2	0.8

Table S4. Energetic data for the top 20 conformations of *trans*-PPV calculated at the M06-2X-D3/6-311+G(d,p) level of theory in chloroform (SMD).

Conformation	Relative enthalpy at zero temperature (kcal/mol)	Relative enthalpy at 298 K (kcal/mol)	Relative free energy at 298 K (kcal/mol)
1	0.3	0.3	0.9
2	0.1	0.1	0.6
3	0.0	0.0	0.2
4	0.1	0.1	0.4
5	REPEAT OF 2	REPEAT OF 2	REPEAT OF 2
6	0.0	0.0	0.2
7	0.1	0.1	0.8
8	0.1	0.1	0.4
9	0.2	0.2	0.8
10	0.0	0.1	0.1
11	0.1	0.2	0.5
12	0.1	0.1	0.6
13	0.1	0.1	0.3
14	0.0	0.0	0.3
15	0.0	0.0	0.6
16	0.0	0.0	0.2
17	0.1	0.1	0.5
18	0.1	0.1	0.0
19	0.2	0.1	0.5
20	0.2	0.1	0.1

Table S5. Energetic data for the top 20 conformations of *cis*-PPV calculated at the CAM-B3LYP-D3/6-311+G(d,p) level of theory in chloroform (SMD).

Conformation	Relative enthalpy at zero temperature (kcal/mol)	Relative enthalpy at 298 K (kcal/mol)	Relative free energy at 298 K (kcal/mol)
1	0.3	0.0	0.9
2	0.1	0.0	0.3
3	0.0	0.0	0.0
4	0.1	0.1	1.4
5	0.0	1.2	1.6
6	0.0	1.2	0.8
7	0.1	0.5	2.3
8	0.1	0.5	2.2
9	0.2	1.1	2.9
10	0.0	1.2	2.2
11	0.1	1.3	2.5
12	0.1	1.5	2.3
13	0.1	2.8	3.0

14	0.0	1.5	1.8
15	0.0	2.4	2.9
16	0.0	1.9	2.2
17	0.1	2.0	1.6
18	0.1	2.4	1.7
19	0.2	2.5	2.1
20	0.2	2.8	1.7

Table S6. Energetic data for the top 20 conformations of *cis*-PPV calculated at the B3LYP-D3/6-311+G(d,p) level of theory in chloroform (SMD).

Conformation	Relative enthalpy at zero temperature (kcal/mol)	Relative enthalpy at 298 K (kcal/mol)	Relative free energy at 298 K (kcal/mol)
1	0.0	0.0	0.6
2	0.0	0.0	0.2
3	0.0	0.0	0.0
4	Failed	Failed	Failed
5	Failed	Failed	Failed
6	1.1	1.3	1.0
7	0.4	0.3	1.7
8	0.4	0.3	1.4
9	1.4	1.4	2.4
10	1.2	1.2	1.9
11	1.1	1.3	1.0
12	1.5	1.5	2.0
13	2.7	2.7	2.7
14	1.9	2.0	2.4
15	2.6	2.5	3.5
16	2.0	2.1	1.8
17	2.1	2.2	1.6
18	2.5	2.6	1.8
19	2.6	2.7	2.1
20	2.8	2.9	1.8

Table S7. Energetic data for the top 20 conformations of *cis*-PPV calculated at the M06-2X-D3/6-311+G(d,p) level of theory in chloroform (SMD).

Conformation	Relative enthalpy at zero temperature (kcal/mol)	Relative enthalpy at 298 K (kcal/mol)	Relative free energy at 298 K (kcal/mol)
1	0.4	0.5	0.2
2	0.3	0.4	0.0
3	0.0	0.0	0.7
4	0.2	0.1	1.2
5	1.5	1.7	1.2
6	1.2	1.5	0.4
7	0.7	0.6	1.1
8	0.8	0.7	1.1
9	1.5	1.6	2.0

10	1.0	1.1	1.6
11	2.1	1.8	3.9
12	3.1	2.6	5.4
13	4.2	4.5	2.5
14	2.3	2.4	2.3
15	2.9	3.1	2.7
16	2.9	3.0	3.5
17	2.8	3.0	2.7
18	3.9	4.1	2.2
19	3.8	4.1	1.9
20	4.0	4.4	1.8

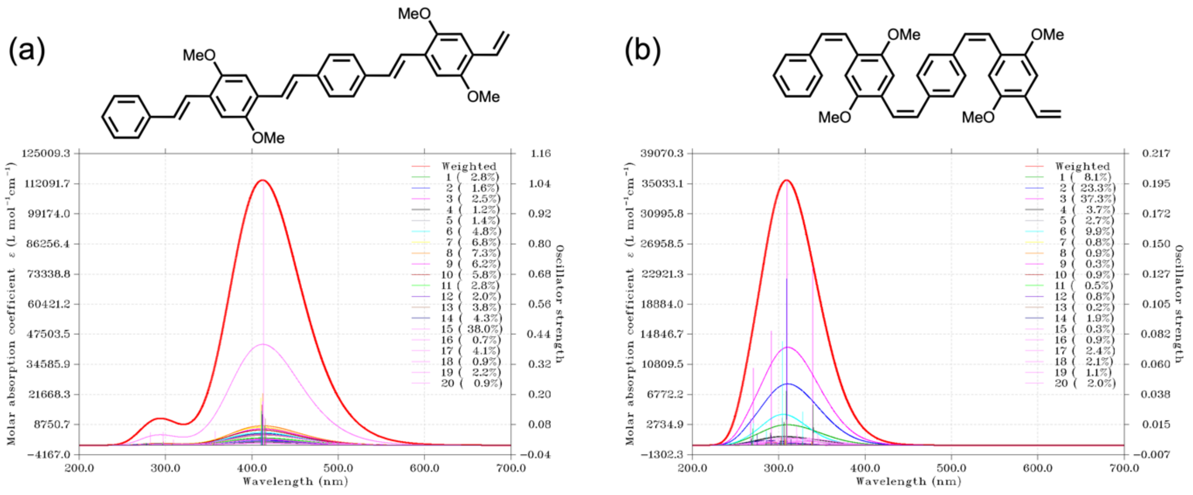

Figure S9. Boltzmann weighted UV-Vis spectra of (a) *trans*- and (b) *cis*-PPV calculated at the CAM-B3LYP/6-311+G(d,p) level of theory in chloroform (SMD).

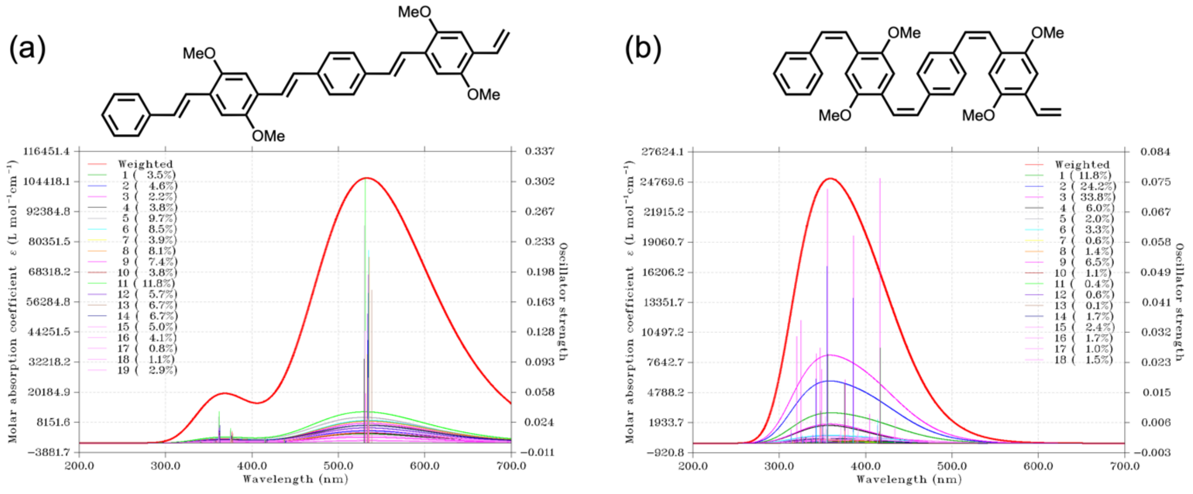

Figure S10. Boltzmann weighted UV-Vis spectra of (a) *trans*- and (b) *cis*-PPV calculated at the B3LYP/6-311+G(d,p) level of theory in chloroform (SMD).

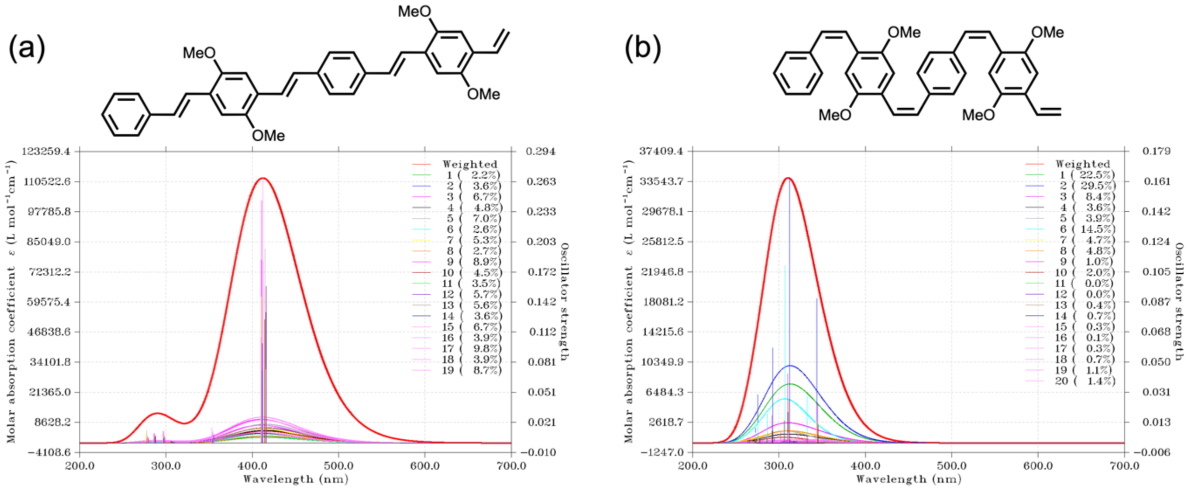

Figure S11. Boltzmann weighted UV-Vis spectra of (a) *trans*- and (b) *cis*-PPV calculated at the M06-2X/6-311+G(d,p) level of theory in chloroform (SMD).

Fragmentation model

In order to understand the magnitude of orbital delocalization the lowest energy conformations for the *trans*- (conformation 15) and *cis*-PPV (conformation 3) optimized at the CAM-B3LYP-D3 level of theory in chloroform (SMD) were taken for analysis. Single point calculations of the *trans*- and *cis*-PPV conformations were performed at the CAM-B3LYP-D3/6-311G(d,p)-SMD($CHCl_3$) level of theory, excluding diffuse functions for further analysis with Multiwfn 3.8.[11] To evaluate the extent of spatial delocalization of the HOMO and HOMO-1 orbitals for *trans*- and *cis*-PPV we used the orbital delocalization index (ODI).[12] ODI is useful in quantifying the spatial delocalization of a particular molecular orbital, where the lower the ODI value corresponds to more orbital delocalization. Intriguingly, while the HOMO (by -0.40 eV) and HOMO-1 (by -0.12 eV) orbitals of *cis*-PPV were lower in energy, less orbital delocalization was observed (Tables S8 – S9).

To discern the change in energy for the HOMO/HOMO-1 orbitals, single point calculations were performed on the individual styrene (1)/(3) and 1,4-dimethoxy-2-vinylbenzene (2)/(4) fragments of *trans*- (Figure S13) and *cis*-PPV (Figure S14). Plotting the HOMO-1, HOMO, LUMO, and LUMO+1 energies of each fragment, a marked decrease in energy was observed in the HOMO/HOMO-1 orbitals *trans*- to *cis*-PPV for the majority of fragments (Figure S15). We determined that HOMO/HOMO-1 energies of the individual fragments led to the decrease in their orbital energy from *trans*- to *cis*-PPV complexes (Table S10). By using the fragmentation analysis, we were able to plot the HOMO and HOMO-1 orbital energies as a function of the dihedral angle with the vinyl group of each fragment (Figures S16-S19). The change in dihedral angles from *trans*- to *cis*-PPV fragments explains the decrease in HOMO and HOMO-1 orbital energies. The dimethoxy-2-vinylbenzene fragment 2 (*trans*-PPV(2) vs. *cis*-PPV(2)) saw the largest decrease in HOMO-1 orbital energy from *trans*- to *cis*-PPV ($\Delta E_{HOMO-1}(2)$ = -0.32 eV) as a result of the 44.2° difference in the dihedral angle (Figure S17). This is due to the twisted conformation that the *cis*-PPV must adopt in the lowest energy conformation.

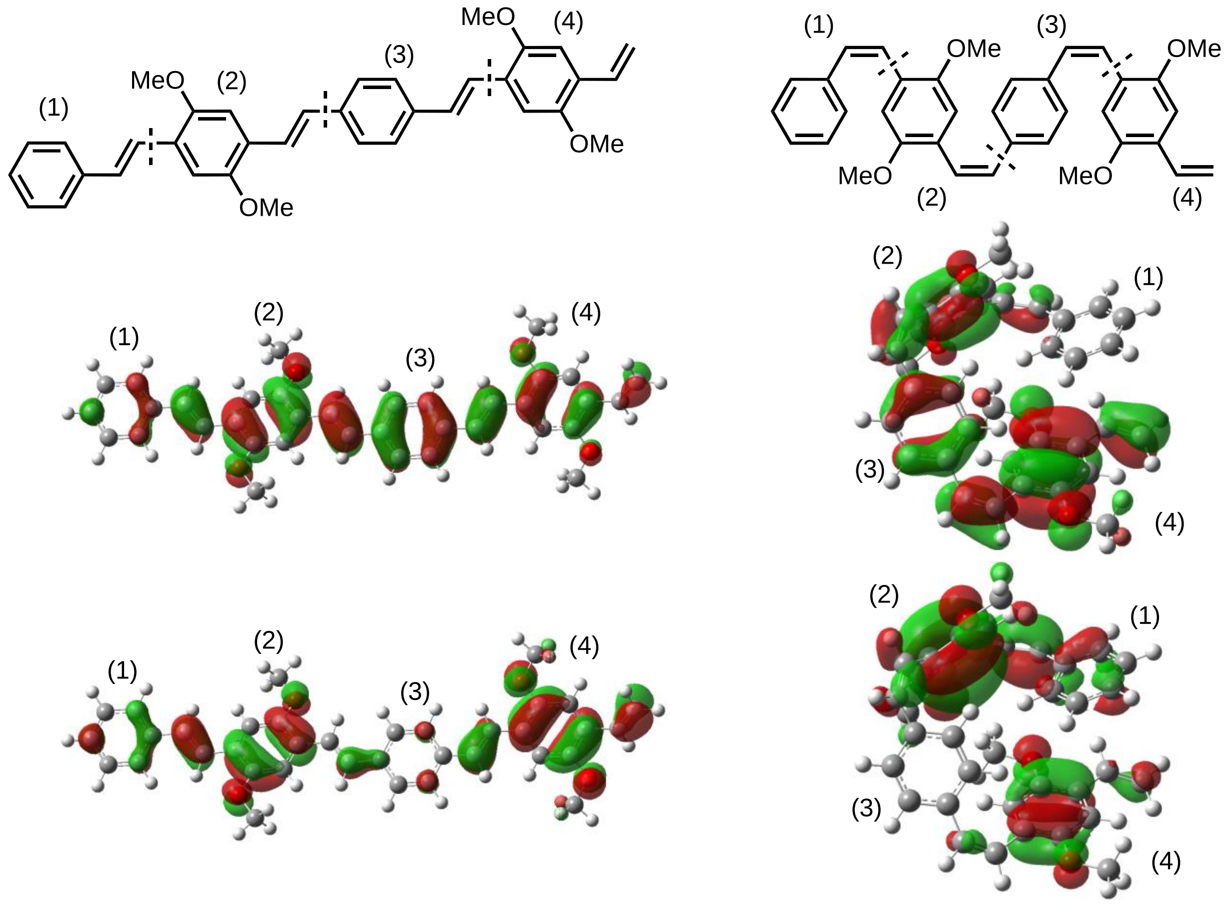

Figure S12. Single point calculations for the HOMO and HOMO-1 orbitals for the *trans*- (left panel) and *cis*-PPV (right panel) calculated at the CAM-B3LYP-D3/6-311G(d,p)-SMD($CHCl_3$) level of theory.

Table S8. Fragment orbital composition analysis for the HOMO/HOMO-1 orbitals for the *trans*-PPV(1), *trans*-PPV(2), *trans*-PPV(3), and *trans*-PPV(4) fragments indicated in Figure S12 calculated at the CAM-B3LYP-D3/6-311G(d,p)-SMD($CHCl_3$) level of theory.

trans-PPV	Energy level (eV)	*trans*-PPV(1)	*trans*-PPV(2)	*trans*-PPV(3)	*trans*-PPV(4)
HOMO	-6.18	12.47%	41.28%	26.87%	19.37%
HOMO ODI	N/A	3.48	3.48	3.48	3.48
HOMO-1	-6.65	12.49%	26.58%	11.15%	49.76%
HOMO-1 ODI	N/A	3.94	3.94	3.94	3.94

Table S9. Fragment orbital composition analysis for the HOMO/HOMO-1 orbitals for the *cis*-PPV(1), *cis*-PPV(2), *cis*-PPV(3), and *cis*-PPV(4) fragments indicated in Figure S12 calculated at the CAM-B3LYP-D3/6-311G(d,p)-SMD($CHCl_3$) level of theory.

cis-PPV	Energy level (eV)	*cis*-PPV(1)	*cis*-PPV(2)	*cis*-PPV(3)	*cis*-PPV(4)
HOMO	-6.58	3.04%	16.72%	21.92%	58.31%

HOMO ODI	N/A	4.63	4.63	4.63	4.63
HOMO-1	-6.78	12.93%	63.93%	2.52%	20.61%
HOMO-1 ODI	N/A	5.34	5.34	5.34	5.34

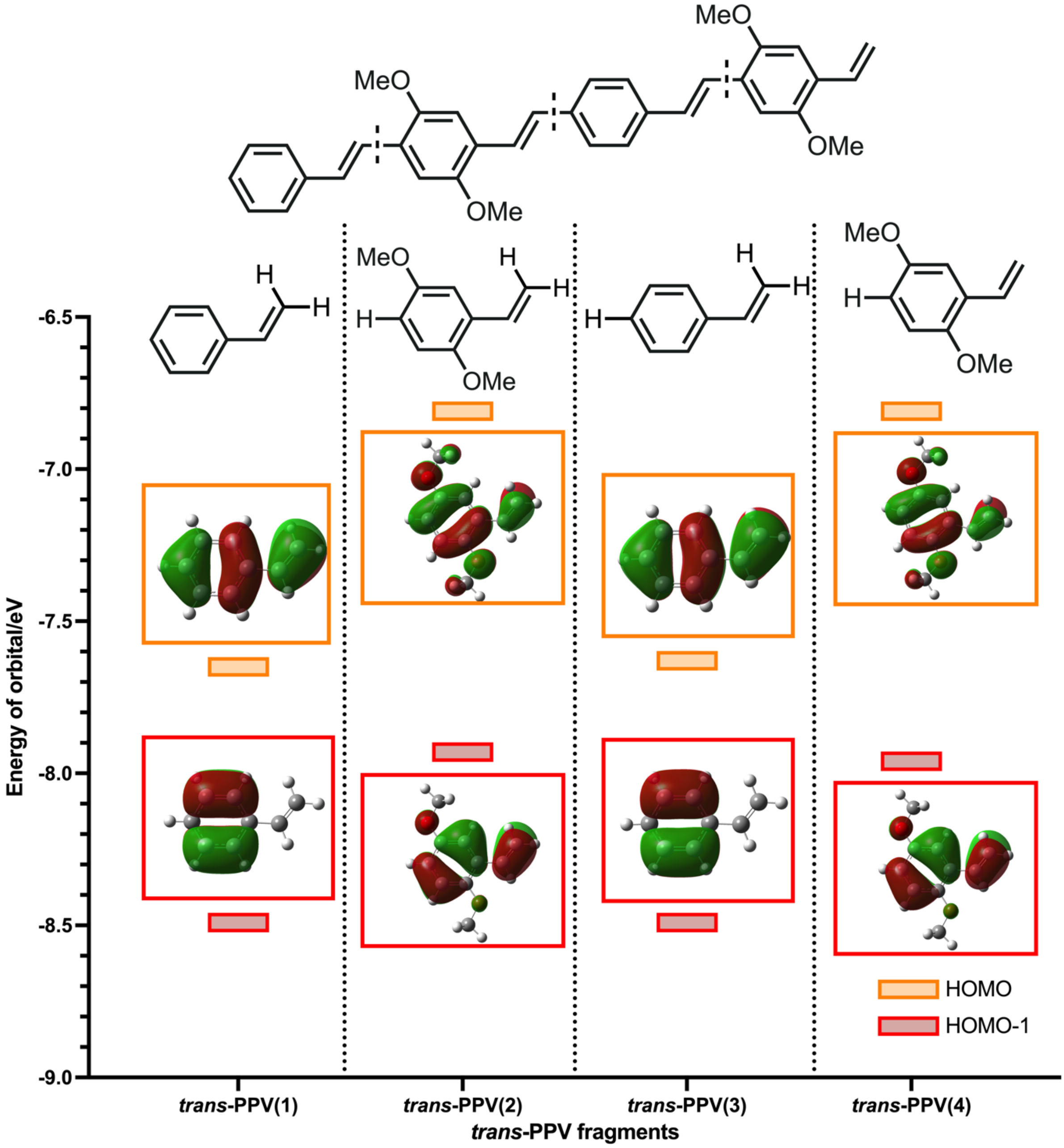

Figure S13. Single point calculations for the HOMO and HOMO-1 orbitals for the *trans*-PPV fragments calculated at the CAM-B3LYP-D3/6-311G(d,p)-SMD($CHCl_3$) level of theory.

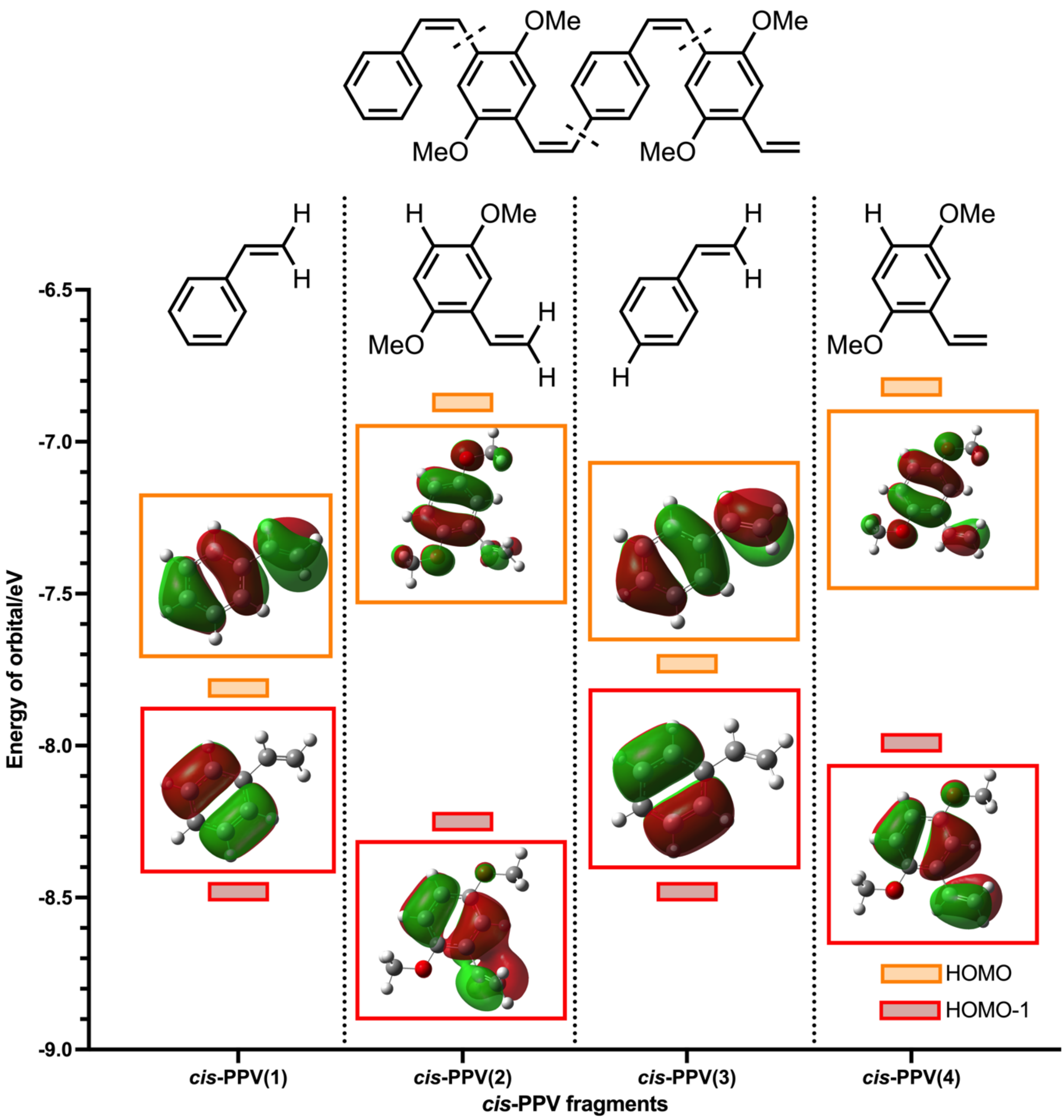

Figure S14. Single point calculations for the HOMO and HOMO-1 orbitals for the *cis*-PPV fragments calculated at the CAM-B3LYP-D3/6-311G(d,p)-SMD($CHCl_3$) level of theory.

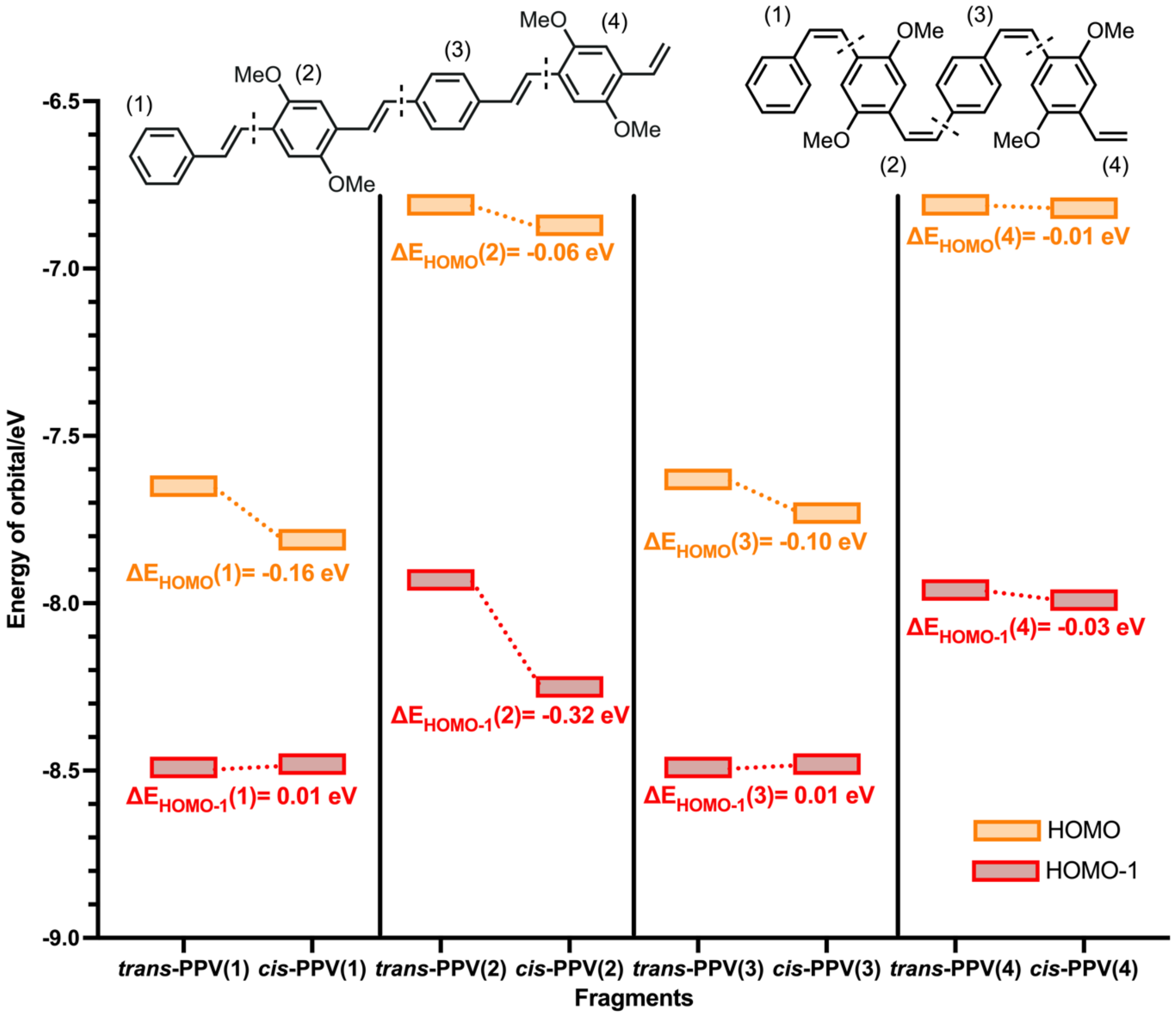

Figure S15. Comparison of the HOMO and HOMO-1 orbitals for the *trans/cis*-PPV fragments calculated at the CAM-B3LYP-D3/6-311G(d,p)-SMD($CHCl_3$) level of theory.

Table S10. Change in energy for the HOMO/HOMO-1 orbitals from the *trans*- to *cis*-PPV fragments calculated at the CAM-B3LYP-D3/6-311G(d,p)-SMD($CHCl_3$) level of theory.

	Fragment 1	Fragment 2	Fragment 3	Fragment 4	Total of all fragments	Full *cis*-/*trans*-PPV
ΔE_{HOMO} (eV)	-0.16	-0.06	-0.10	-0.01	-0.33	-0.40
ΔE_{HOMO-1}	0.01	-0.32	0.01	-0.03	-0.33	-0.12

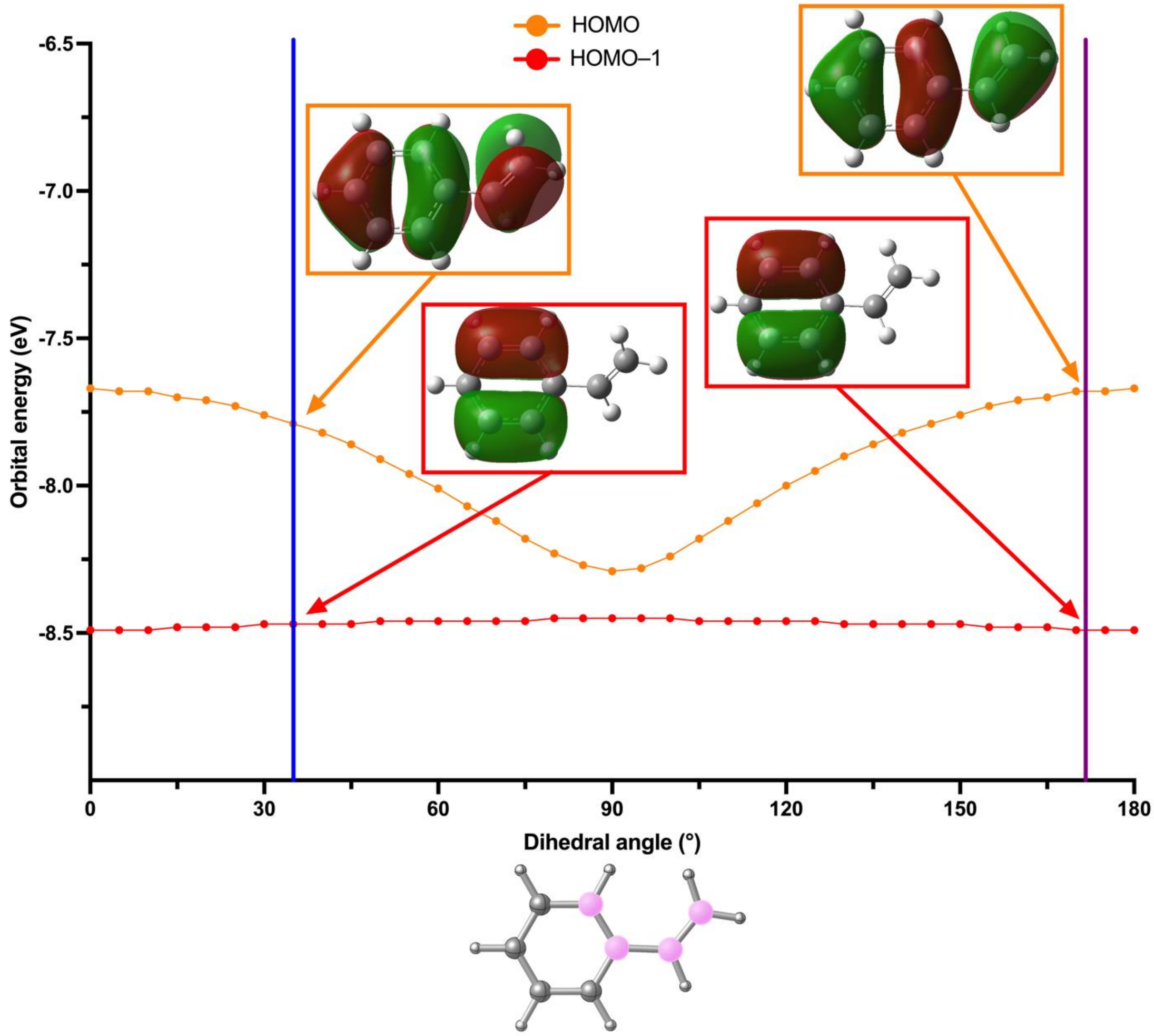

Figure S16. Plot of the HOMO and HOMO-1 orbitals with respect to the dihedral angle of fragment 1 where the blue line represents the dihedral angle of *cis*-PPV and the purple line represents the dihedral angle of *trans*-PPV calculated at the CAM-B3LYP-D3/6-311G(d,p)-SMD($CHCl_3$) level of theory

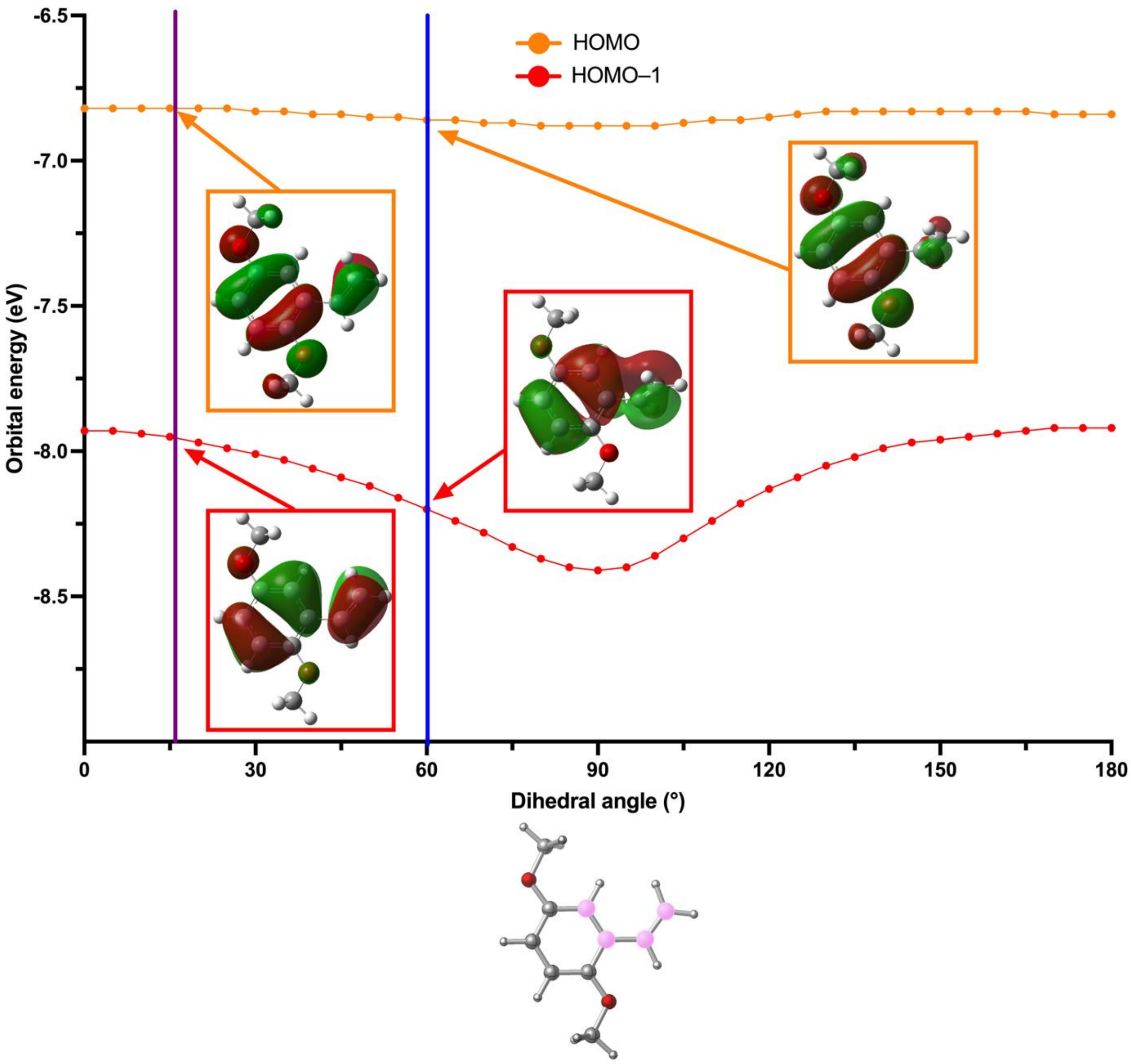

Figure S17. Plot of the HOMO and HOMO-1 orbitals with respect to the dihedral angle of fragment 2 where the blue line represents the dihedral angle of *cis*-PPV and the purple line represents the dihedral angle of *trans*-PPV calculated at the CAM-B3LYP-D3/6-311G(d,p)-SMD($CHCl_3$) level of theory

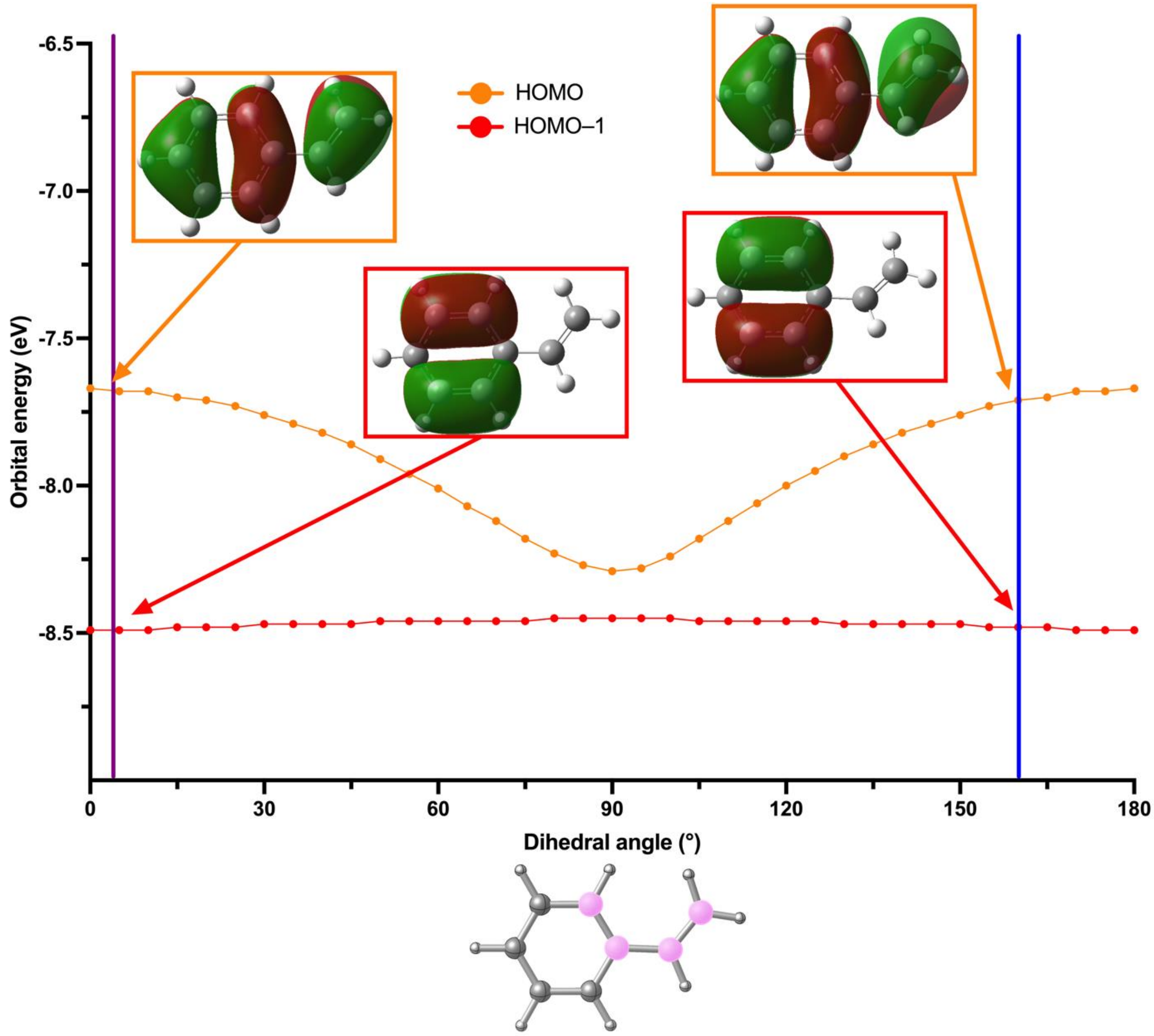

Figure S18. Plot of the HOMO and HOMO-1 orbitals with respect to the dihedral angle of fragment 3 where the blue line represents the dihedral angle of *cis*-PPV and the purple line represents the dihedral angle of *trans*-PPV calculated at the CAM-B3LYP-D3/6-311G(d,p)-SMD($CHCl_3$) level of theory

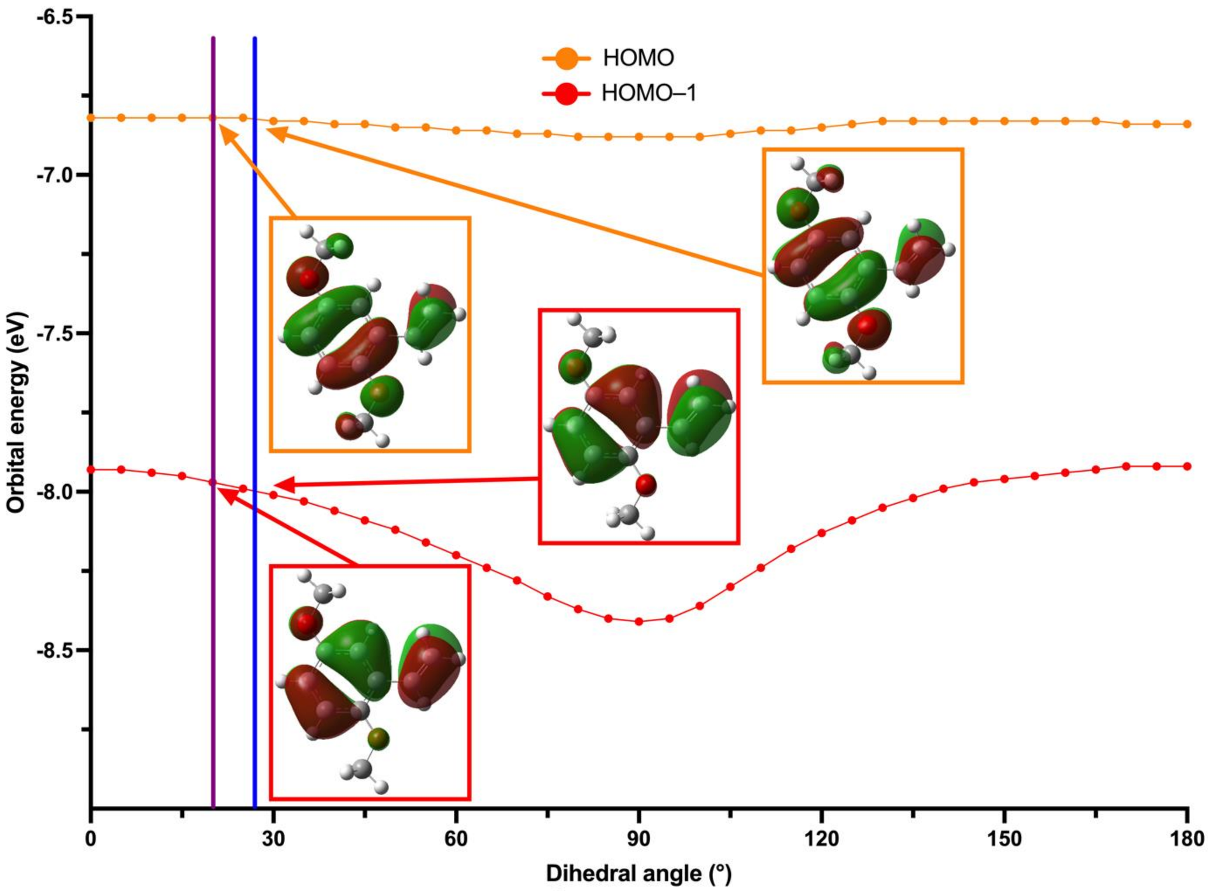

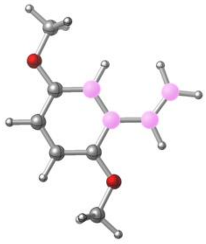

Figure S19. Plot of the HOMO and HOMO-1 orbitals with respect to the dihedral angle of fragment 4 where the blue line represents the dihedral angle of *cis*-PPV and the purple line represents the dihedral angle of *trans*-PPV calculated at the CAM-B3LYP-D3/6-311G(d,p)-SMD($CHCl_3$) level of theory

Steady-state absorption and emission

The absorption and emission measurements were carried out in dilute chloroform solutions. Absorption spectra were collected on a Shimadzu UV-2600 UV-visible spectrophotometer using 1.0 cm path length quartz cuvettes. Fluorescence measurements were collected on a Horiba FluoroMax 4 spectrofluorometer with entrance and exit slits set to 2.0 nm and an integration time of 0.1 s.

Two-Photon Absorption (TPA)

The TPA cross-section of liquid samples were determined using the two-photon induced fluorescence technique employing two-photon power-dependent fluorescence intensity. TPA was performed with a mode-locked Ti:Sapphire laser (Spectra-Physics Millenia) adjustable from 760 to 830 nm, producing 110 fs output pulses at an 80 MHz repetition rate. For the current investigation, all liquid samples contained in a 1 cm quartz cuvette were excited at 800 nm and generated fluorescence collected in the perpendicular direction to the incident beam using a Hamamatsu photomultiplier tube and photocounting unit. The emission detection wavelengths were set to be 520 nm for poly-*cis*-**1**-*b*-*cis*-**2** and poly-*cis*-**1**-*b*-*trans*-**2** by Newport Oriel Cornerstone monochromator. Using a neutral density filter, the input power was varied, and the TPA cross-sections were calculated by comparing with a known standard solution. For this measurement, we used Coumarin 307 (δ = 28)[13] standard solution in methanol as a reference.

Time-Resolved Fluorescence Up-Conversion.

The detailed experimental setup used for the measurements of femtosecond fluorescence upconversion previously described by Goodson and co-workers was used in this study.[14] The study of fluorescence upconversion for poly-*cis*-**1**-*b*-*cis*-**2** and poly-*cis*-**1**-*b*-*trans*-**2** were performed at an up-converted wavelength of 346.7 nm using a FOG- 100 system (CDP

Instruments, Inc.). A mode-locked Ti:Sapphire (Tsunami, Spectra Physics) laser source of 800 nm (~ 700 mW) was used as a fundamental beam. The fundamental beam was frequency doubled by passing it through a β-barium borate (BBO) nonlinear crystal. By second harmonic generation, 400 nm light was produced which acted as the excitation source for the sample in solution phase (1 mm thick rotating sample cell). The remaining 800 nm gate beam was directed to a gold-coated retroreflector mirror connected to a 2 ns optical delay line, before being focused together with the fluorescence on a 0.5 mm Type I BBO crystal. The angle of the crystal was adjusted to the phase-matching conditions at the fluorescence wavelength of interest. The upconverted signal which induced a delay in time by adjusting the path length of the gate beam. Both the gate and the sample fluorescence passed through a BBO crystal. Sum frequency generation occurred when the gate pulse entered the BBO crystal at the same time as the sample fluorescence. As delay is introduced to the gate beam, fluorescence decay can be detected. The detected (up-converted) light is collected by a monochromator and a photomultiplier tube (R1527P, Hamamatsu).

Femtosecond Transient Absorption

The femtosecond transient experimental setup contained a Spectra-Physics ultrafast amplified laser system. The Spectra-Physics ultrafast amplified laser system contained a Millennia Pro laser (continuous wave at 532nm with a power of 4.3W) that was used to pump Spectra-Physics Tsunami Ti:Sapphire mode-locked laser (pulsed at 790nm, output power: 250mW, repetition rate: 80MHz, approximate pulse width: 80fs) and Empower Q-switched laser (pulsed at 527nm, output power: 8W, repetition rate: 1kHz, approximate pulse width: 200ns) were used to pump a Spitfire Amplifier laser to generate an amplified 790nm output beam with: an approximate pulse width of 110 fs; repetition rate of 1kHz; and approximate output power of 1W. The 790nm amplified femtosecond pulse was directed into a beamsplitter, where approximately 15% of the pulse was used to generate the probe beam for fs-TA

experiment and other roughly 85% was used to pump a Spectra-Physics Optical Parametric Amplifier (OPA). After the beamsplitter and OPA, both probe and pump beams were directed into an Ultrafast System Inc. Helios fs-TA optical setup. Specifically, the pump beam was directed into an optical chopper and focused onto samples in a quartz cuvette with a 2mm path length. The samples were liquid solutions of PPV prepared in chloroform and had an optical density ranging from 0.5–0.8 for experiments. The probe beam was directed through a computer-controlled delay line and focused onto a 3mm sapphire plate that generated a white light continuum. The probe beam/white light continuum and sample signal was directed and focused onto an Ocean Optics spectrometer and charge-coupled device (CCD), which collected the difference in absorbance signal (ΔA) as a result of the pump being on versus off due to the optical chopper. The Helios software, by Ultrafast Systems Inc., was used for data acquisition and the data analysis was done using Surface Xplorer and Origin Pro software.

References

1. Hancock, S. N,; Yuntawattana, N.; Valdez, S. M.; Michaudel, Q. Expedient synthesis and ring-opening metathesis polymerization of pyridinonorbornenes. *Polym. Chem.* **2022**, *12,* 5530–5535.
2. Padron, S.; Patlan, R.; Gutierrez, J.; Santos, N.; Eubanks, T.; Lozano, K. Production and Characterization of Hybrid BEH-PPV/PEO Conjugated Polymer Nanofibers by Forcespinning^{TM}. *J. Appl. Polym. Sci.* **2012**, *125,* 3610–3616.
3. Lidster, B. J.; Kumar, D. R.; Spring, A. M.; Yu, C.-Y.; Helliwell, M.; Raftery, J.; Turner, M. L. Alkyl substituted [2.2]paracyclophane-1,9-dienes. *Org. Biomol. Chem.* **2016**, *14,* 6079–6087.
4. Lidster, B. J.; Behrendt, J. M.; Turner, M. L.; Monotelechelic poly(*p*-phenylenevinylene)s by ring opening metathesis polymerization. *Chem. Commun.* **2014**, *50*, 11867–11870.
5. Würth, C.; Grabolle, M.; Pauli, J.; Spieles, M.; Resch-Genger, U. Relative and absolute determination of fluorescence quantum yields of transparent samples. *Nat. Protoc.* **2013**, *8*, 1535–1550.
6. Harder, E.; Damm, W.; Maple, J.; Wu, C.; Reboul, M.; Xiang, J. Y.; Wang, L.; Lupyan, D.; Dahlgren, M. K.; Knight, J. L.; Kaus, J. W.; Cerutti, D. S.; Krilov, G.; Jorgensen, W. L.; Abel, R.; Friesner, R. A. OPLS3: A Force Field Providing Broad Coverage of Drug-like Small Molecules and Proteins. *J. Chem. Theory Comput.* **2016**, *12*, 281–296.
7. (a) Yanai, T.; Tew, D. P; Handy, N. C. A new hybrid exchange–correlation functional using the Coulomb-attenuating method (CAM-B3LYP). *Chem. Phys. Lett.* **2004**, *393*, 51–57. (b) Becke, A. D. Density-functional thermochemistry. III. The role of exact exchange. *J. Chem. Phys.* **1993**, *98*, 5648–5652. (c) Kim, K.; Jordan, K. D. Comparison of Density Functional and MP2 Calculations on the Water Monomer and Dimer. *J. Phys. Chem.* **1994**, *98*, 10089–10094. (d) Stephens, P. J.; Devlin, F. J.; Chabalowski, C. F.; Frisch, M. J. Ab Initio Calculation of Vibrational Absorption and Circular Dichroism Spectra Using Density Functional Force Fields. *J. Phys. Chem.* **1994**, *98*, 11623–11627. (e) Grimme, S.; Antony, J.; Ehrlich, S.; Krieg, H. A consistent and accurate ab initio parameterization of density functional dispersion correction (DFT-D) for the 94 elements H-Pu. *J. Chem. Phys*. **2010**, *132*, 154104. (f) Clark, T.; Chandrasekhar, J.; Spitznagel, G. W.; Schleyer, P. V. R. Efficient diffuse function-augmented basis sets for anion calculations. III. The 3-21+G basis set for first-row elements, Li-F. *J. Comput. Chem*. **1983**, *4*, 294–301. (g) Krishnan, R., Binkley, J. S., Seeger, R., Pople, J. A. Self-consistent molecular orbital methods. XX. A basis set for correlated wave functions. *J. Chem. Phys.* **1980**, *72*, 650–654. (h) Miertuš, S.; Scrocco, E.; Tomasi, J. Electrostatic interaction of a solute with a continuum. A direct utilization of AB initio molecular potentials for the prevision of solvent effects. *Chem. Phys.* **1981**, *55*, 117–129. (i) Miertuš, S.; Tomasi, J. Approximate evaluations of the electrostatic free energy and internal energy changes in solution processes. *Chem. Phys.* **1982**, *65*, 239–245. (j) Pascual-ahuir, J. L.; Silla, E.; Tuñon, I. GEPOL: An improved description of molecular surfaces. III. A new algorithm for the computation of a solvent-excluding surface. *J. Comput. Chem.* **1994**, *15*, 1127–1138.
8. **Schrödinger Release 2023-2**: MacroModel, Schrödinger, LLC, New York, NY, 2023.
9. Frisch, M. J.; Trucks, G. W.; Schlegel, H. B.; Scuseria, G. E.; Robb, M. A.; Cheeseman, J. R.; Scalmani, G.; Barone, V.; Petersson, G. A.; Nakatsuji, H.; Li, X.; Caricato, M.; Marenich, A. V.; Bloino, J.; Janesko, B. G.; Gomperts, R.; Mennucci, B.; Hratchian, H. P.; Ortiz, J. V.; Izmaylov, A. F.; Sonnenburg, J. L.; Williams-Young, D.; Ding, F.; Lipparini, F.; Egidi, F.; Goings, J.; Peng, B.; Petrone, A.; Henderson, T.; Ranasinghe,

D.; Zakrzewski, V. G.; Gao, J.; Rega, N.; Zheng, G.; Liang, W.; Hada, M.; Ehara, M.; Toyota, K.; Fukuda, R.; Hasegawa, J.; Ishida, M.; Nakajima, T.; Honda, Y.; Kitao, O.; Nakai, H.; Vreven, T.; Throssell, K.; Montgomery, J. A., Jr.; Peralta, J. E.; Ogliaro, F.; Bearpark, M. J.; Heyd, J. J.; Brothers, E. N.; Kudin, K. N.; Staroverov, V. N.; Keith, T. A.; Kobayashi, R.; Normand, J.; Raghavachari, K.; Rendell, A. P.; Burant, J. C.; Iyengar, S. S.; Tomasi, J.; Cossi, M.; Millam, J. M.; Klene, M.; Adamo, C.; Cammi, R.; Ochterski, J. W.; Martin, R. L.; Morokuma, K.; Farkas, O.; Foresman, J. B.; Fox, D. J. Gaussian 16, Rev. A.03, Gaussian, Inc.: Wallingford CT, 2016.

10. (a) Zhao, Y.; Truhlar, D. G. The M06 Suite of Density Functionals for Main Group Thermochemistry, Thermochemical Kinetics, Noncovalent Interactions, Excited States, and Transition Elements: Two New Functionals and Systematic Testing of Four M06- Class Functionals and 12 Other Function. *Theor. Chem. Acc.* **2008**, *120*, 215–241. (b) Zhao, Y.; Truhlar, D. G. Density Functionals with Broad Applicability in Chemistry. *Acc. Chem. Res.* **2008**, *41*, 157–167.
11. Lu, T.; Chen, F. Multiwfn: A Multifunctional Wavefunction Analyzer. *J. Comput. Chem.* **2012**, *33*, 580−592.
12. Lu, T.; Chen F. Calculation of Molecular Orbital Composition. *Acta Chimica Sinica* **2011**, *69*, 2393–2406.
13. Melnikov, S. A.; Serdobintsev, Yu. P.; Vedyaykin, D. A.; Khodorkovskii, A. M. Two-photon absorption cross section for Coumarins 102, 153 and 307. *J. Phys.: Conf. Ser.* **2017**, *917*, 062029.
14. Ricci, F.; Mandal, H.; Wajahath, M.; Burdick, R.; Villabona-Monsalve, J. P.; Hussain, S.; Goodson, III. T. Investigations of Coherence in Perovskite Quantum Dots with Classical and Quantum Light. *J. Phys. Chem. C* **2023**, *127*, 3579–3593.
